# Phthalocyanines: An Old Dog Can Still Have New (Photo)Tricks!

**DOI:** 10.3390/molecules26092823

**Published:** 2021-05-10

**Authors:** Andrea M. Schmidt, Mário J. F. Calvete

**Affiliations:** 1 LifeEstetika, Laser Solutions, Universitätstadt Tübingen, Maria-von-Linden Strasse, 72076 Tübingen, Germany; andrea.m.schmidt.dls@gmail.com; 2 University of Coimbra, CQC, Department of Chemistry, Rua Larga, 3004-535 Coimbra, Portugal

**Keywords:** phthalocyanine, photonics, photoelectronics, photovoltaics, photocatalysis, charge transfer, nonlinear optics

## Abstract

Phthalocyanines have enjoyed throughout the years the benefits of being exquisite compounds with many favorable properties arising from the straightforward and diverse possibilities of their structural modulation. Last decades appreciated a steady growth in applications for phthalocyanines, particularly those dependent on their great photophysical properties, now used in several cutting-edge technologies, particularly in photonic applications. Judging by the vivid reports currently provided by many researchers around the world, the spotlight remains assured. This review deals with the use of phthalocyanine molecules in innovative materials in photo-applications. Beyond a comprehensive view on the recent discoveries, a critical review of the most acclaimed/considered reports is the driving force, providing a brief and direct insight on the latest milestones in phthalocyanine photonic-based science.

## 1. Introduction

Phthalocyanines (Pcs) are highly conjugated and essentially planar aromatic macrocycles, consisting of four iminoisoindoline units, containing 18 delocalized π-electrons and widely praised for their thermal and chemical stability. Such electronic delocalization permits Pcs to display intense absorption bands in the near infrared region of the electronic spectrum, reaching very high extinction coefficients (~10^5^ M^−1^ cm^−1^), accompanied by high fluorescence quantum yields, concomitantly with favorable redox activity and rich electrochemistry [1,2,3,4,5].

**Figure 1:**
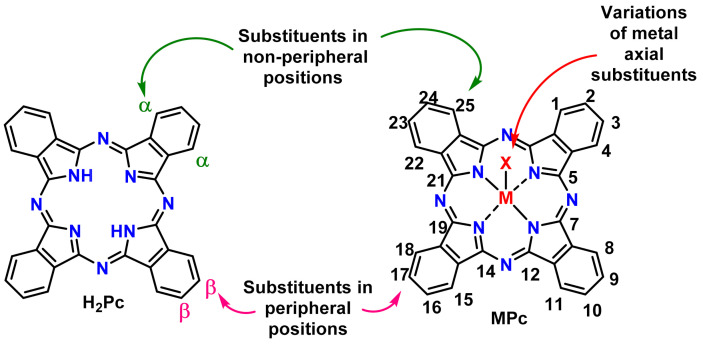
General structure of metal-free phthalocyanine (**left**) and metal phthalocyanine complex (**right**), with schematic representation of possible structural modulation sites and ring numbering.

Phthalocyanines display great structural flexibility, existing both as free bases (metal-free) and in the form of metal complexes, hosting ca. 70 different elements in the central cavity, some of them allowing structural modulation via functionalization with metal-axial ligands. In addition, a substantial variety of substituents can be incorporated into the phthalocyanine core at positions designated as α (non-peripheral) and as β (peripheral) (Figure 1), allowing for the design of desired electronic and/or solubilizing properties, depending upon the application [5,6].

Beyond their absorption features, many other recognized properties arose from the preparation of Pcs, allowing these macrocycles to be considered leading chemical entities, applied in several technologies over the years, including as catalysts [7,8,9,10], sensors [11,12,13], thin films [14,15,16,17], liquid crystals [18,19,20], semi-conductors [21,22,23], textile dyes [24,25], light-harvesting dyes in solar cells/organic photovoltaics [26,27,28,29], absorbers in nonlinear optics [30,31,32,33,34,35,36], photocatalysts [37,38], photosensitizers in phototherapies [39,40], photosensitizers photodynamic inactivation of bacteria [41,42], and as contrast agents in medical imaging [43,44,45,46].

Of the many applications found for phthalocyanines over the many decades after their discovery [47,48,49], recent years witnessed a solid growth in applications for phthalocyanines, particularly those relying on their excelling photophysical properties, and to the technological instrument development that permitted phthalocyanines to be used in several cutting-edge technologies, particularly in photonic applications. The intrinsic phthalocyanines photo-properties allowed this family of tetrapyrrolic macrocycles to emerge as prominent molecules, demonstrating how an “old” compound can still demonstrate groundbreaking applicability.

In this review, the focus is directed to the use of phthalocyanine molecules exclusively in innovative materials in photo-applications. Aware of the importance of phthalocyanine-related molecules, such as subphthalocyanines, naphthalocyanines, porphyrazines, and porphyrinoids, the recent discoveries (in the last five years) will solely consider the application of phthalocyanines in photonic-based materials technologies. Indeed, there are several highly cited review records in several sub-fields, e.g., photomedicine [50,51,52,53,54,55,56,57,58,59,60], photovoltaics [61,62,63], nonlinear optics [64,65,66], photocatalysis [67,68,69], among other photo-applications [70,71,72], portraying phthalocyanines among the therein discussed materials/molecules. Herein, more than a comprehensive view on the recent discoveries, a critical review of the most acclaimed/considered reports (i.e., most cited) was the driving force in this work, aiming to provide a brief and direct insight on the latest milestones in phthalocyanine science.

## 2. Bibliometric Analysis

To explore the research status on this area of study, a short bibliometric analysis was performed by assessing the publication trends from selected research area outputs [73,74]. The search strings (phthalocyanin*) and (phthalocyanin* AND photo*) present in the title and/or abstract and/or keywords were used in two well-known engines, Scopus collection and Web of Science Core Collection (WoS). Since the latter retrieved a larger number of records (Figure S1, Supplementary Materials), this was the data used to establish the conclusions regarding the production of new reports on the field.

**Figure 2:**
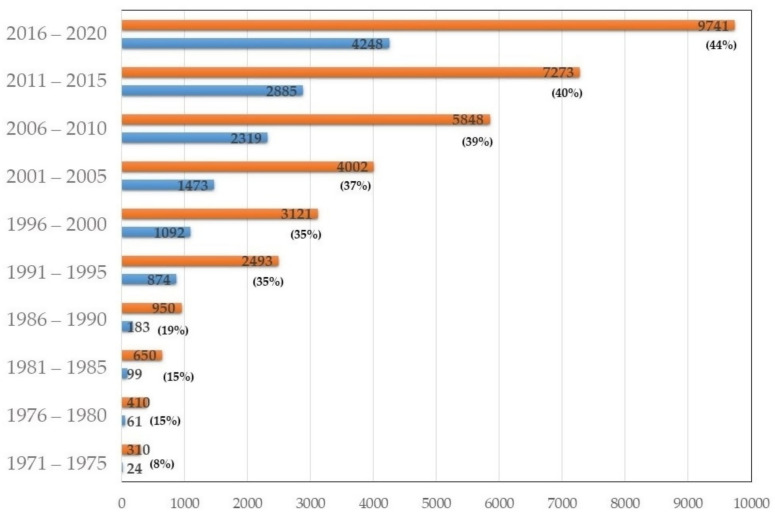
Relative weight of reports retrieved using the term “phthalocyanin*” (orange bars) vs. its refining with the term “photo*” (blue bars). In parentheses are the percentage weights of photo-applications over all others.

Using the WoS search engine, the results were separated in five-year spans, in order to better establish possible trends (Figure 2). In a first look at the graphic, the sound increase in general publications in phthalocyanines stands out. Naturally, the photo-applications using these macrocycles accompany this trend, showing their prominence within the phthalocyanine field. The appearance of new equipment dedicated to study a vast array of photo-processes naturally boosted their applicability in the 1990s; nevertheless, the steady increase is remarkable. This demonstrates the lively interest that this sub-field brings into the phthalocyanine field.

In the Web of Science retrieval of articles contained in title, abstract, or keywords, the term “phthalocyanin*” further refined with the term “photo*”, from 1971 to 2020 (50 years), allowed the establishment of a tendency throughout the years. While increasing the number of publications on photo-applications of phthalocyanines (see Figure 2), the weight of each country in new reports has evolved (see Figure S2). The USSR weight in the beginning of the 1970s (continuing form earlier years) was replaced by North American countries (USA and Canada) in subsequent decades (until 1990). Until 2005, Japan also contributed largely to publication records, as well as European countries, such as England, Germany (including the extinct Federal Republic of Germany), and France. It was only at this time that the Peoples Republic of China claimed its prominent stand in the number of reports provided to the scientific community. In a relatively specialized field such as the synthesis and application of phthalocyanines, several countries stand out due to some prolific research groups, as was the case of Canada in the 1980s, or more recently Turkey and South Africa. Overall, favorable research policies and adequate funding opportunities represent what drive the success of the phthalocyanine field.

## 3. Materials Photo-Applications of Phthalocyanines

### 3.1. Photovoltaics

In light harvesting applications, Pcs hold a prominent position, acting first as antennas, since they absorb light very efficiently in the visible region of the solar spectrum, and, second, once photoexcited, they act as an electron donor for the acceptor moiety. Pcs are also good organic semiconducting chromophores, showing a prolonged photoresponse in the 600–800 nm range, where the photon flux is maximum, further displaying high charge-carrier mobilities and notable exciton diffusion lengths [75,76].

**Figure 3:**
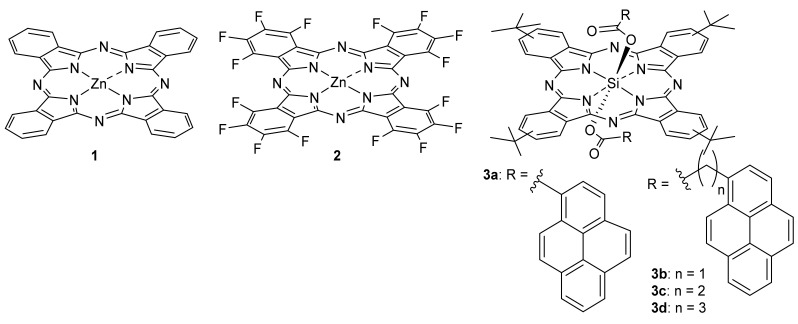
Pcs used in photovoltaic ternary systems.

For instance, Leo and collaborators applied band structure engineering to tune the band gap and band-edge energies [77]. Empirically combining different inorganic semiconductors was a strategy to reach such purposes by blending materials with different energy levels [78], but this approach in organic semiconductors was prevented by the strong localization of the electronic states in these materials. The authors circumvented this issue by considering long-range Coulomb interactions, which enable continuous tuning in blends [79]. They studied a ternary bulk heterojunction of Pcs **1** and **2** (Figure 3) as donors with C60 as an acceptor and demonstrated by photoelectron spectroscopy the continuous tuning of the ionization energies of Pc thin films by blending Pc **1** with their halogenated derivatives, herein Pc **2**. The mixing ratio of Pc **2** and Pc **1** was varied from pure Pc **2** to pure Pc **1** while the C60 content was fixed at 60 weight percent. This in turn allows continued tuning of the photovoltaic gap and open-circuit voltage of organic solar cells.

Ke and Ameri reported a polymer/fullerene-based bulk heterojunction solar cell with extended absorption bandwidth by blending axially substituted silicon phthalocyanines (Pcs **3a**–**d**) (Figure 3 [80]. The authors used a system based on poly(3-hexylthiophene) (P3HT) and phenylC61-butyric acid methyl ester (PC61BM) as the electron donor and acceptor, respectively. The influence of the linker size on cell efficiency was studied, reaching a power conversion efficiency (PCE) of 4.14% bearing a 20% weight content of phthalocyanine when Pc **3d** was used. The authors attributed this higher efficiency of the solar cell containing the Pc dye with a longer linker to weaker intramolecular interactions as well as the higher solubility, which otherwise would cause aggregation.

**Figure 4:**
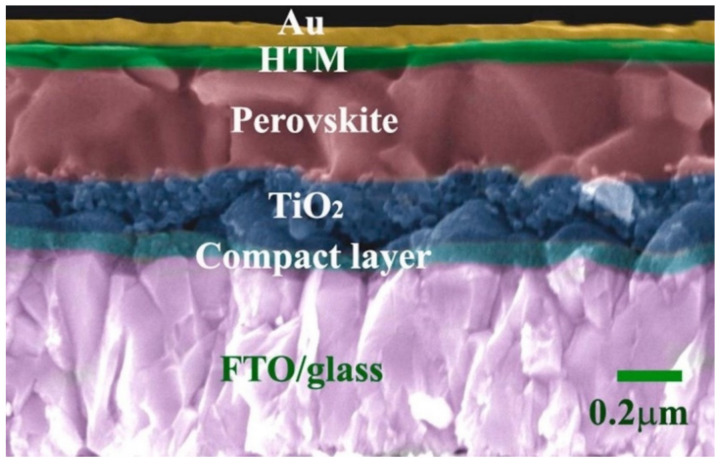
Representative cross-sectional image of a device based on phthalocyanine as HTM. Adapted with permission from ref [89]. Copyright 2017 Elsevier.

Perovskite materials emerged recently, most prominently as components of solar cells. Synthetic perovskites are recognized as potential inexpensive base materials for high-efficiency commercial photovoltaics. These materials have unique photoelectric properties, being suitable for light harvesting in photovoltaics, e.g., appropriate and adjustable band gap [81], ambipolar charge transport [82], and long carrier diffusion length [83]. However, several issues remain to be addressed, namely the instability of Perovskite solar cells (PSC) [84,85,86]. One strategy is the use of improved hole-transporting materials (HTM), which may enhance both the long-time stability and performance of PSCs [87,88]. In this respect, phthalocyanines appeared as ideal partners due to their already highly suitable properties for solar cells. Figure 4 depicts a cross-sectional image of a typical perovskite solar cell device. It consists of a gold electrode, a HTM of a phthalocyanine, a light absorber of perovskite, and a compact (dense) TiO_2_ (dTiO_2_) layer, deposited with a FTO/glass.

**Figure 5:**
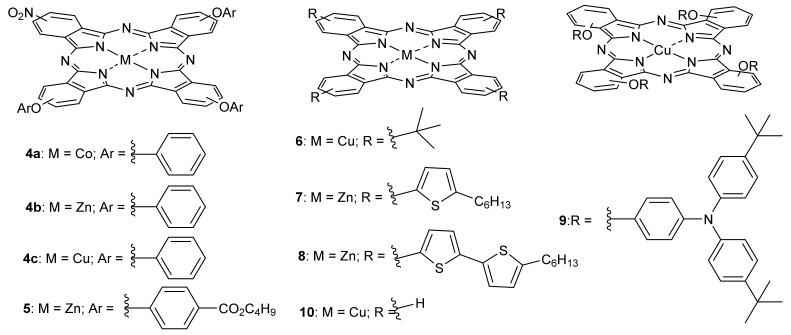
Pcs used in perovskite solar cells.

In recent years, several reports received merited attention, where some Pcs were used as HTMs in perovskite solar cells (Figure 5). Table 1 shows the photovoltaic parameters obtained for those PSCs employing Pcs as HTMs. The short-circuit current density J_SC_, which can be obtained from the standard 100 mW/cm^2^ solar spectrum (AM1. 5), is determined by optical losses, that is, by considering that photons from a part of the spectrum are either not absorbed in the solar cell or are absorbed without generating electron–hole pairs. The short-circuit current is the current through the solar cell when the voltage across the solar cell is zero (i.e., when the solar cell is short circuited). The open-circuit voltage, V_OC_, is the maximum voltage available from a solar cell, and this occurs at zero current [63]. The open-circuit voltage corresponds to the amount of forward bias on the solar cell due to the bias of the solar cell junction with the light-generated current. The short-circuit current and the open-circuit voltage are the maximum current and voltage, respectively, from a solar cell. However, at both of these operating points, the power from the solar cell is zero. The “fill factor”, “FF”, is a parameter which, in conjunction with V_OC_ and J_SC_, determines the maximum power from a solar cell. Finally, the efficiency is the most commonly used parameter to compare the performance of one solar cell to another. Efficiency is defined as the ratio of energy output from the solar cell to input energy from the sun [63].

For instance, Zhang and collaborators reported a small family of non-symmetrically substituted phthalocyanines (Pcs **4a**–**c** and **5**, Figure 5), synthesized to be used as HTM in PSCs [89,90,91]. Tetraphenoxy-substituted metallophthalocyanines **4a**–**c** were studied (entries 1–3, Table 1), and Zn as the central moiety was found to show higher performance with a PCE = 14.35%. Hence, having Zn as a central metal, the same authors additionally compared it with Pc **5** (entry 4, Table 1), a phthalocyanine bearing four peripheral oxyphenyl butylester groups, which further increased PCE to 15.74% [91].

**Figure 6:**
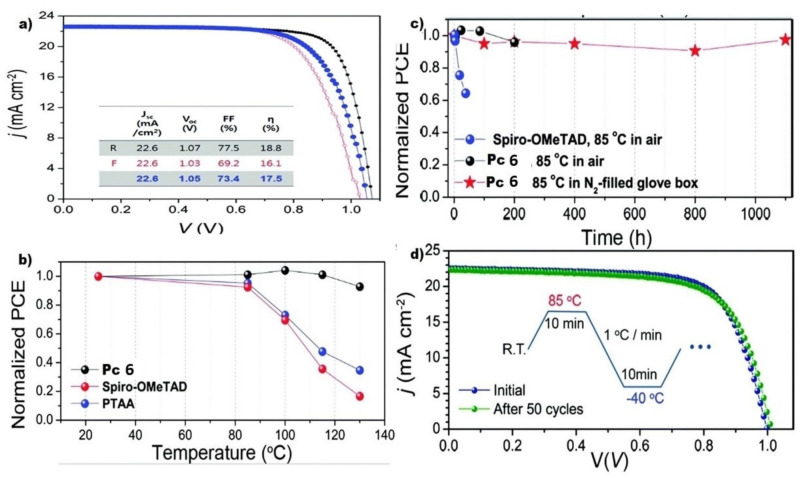
Cell performance and thermal stability. (**a**) J–V curves for the best cell using Pc **6** as HTM (in reverse (black) and forward (red) scans). The photovoltaic parameters of the best cell are shown in the inset table (average values in blue); (**b**) Stability of the devices tested at different temperatures (25, 85, 100, 115, and 130 °C, respectively) for 30 min, employing Pc **6** (black color), spiro-OMeTAD (red color), and PTAA (blue color); (**c**) Long-term stability of the devices stressed at 85 °C in air (25–30% humidity), using Pc **7** (black color) and spiro-OMeTAD (blue color) and 1100 h stability of the device utilizing Pc **6** at 85 °C in a nitrogen-filled glove box (red color); (**d**) J–V curves of a Pc **6** device before and after the thermal cycling test of 50 cycles within the temperature range between −40 °C and 85 °C. Adapted with permission from ref [92]. Copyright 2018 The Royal Society of Chemistry.

Along with HTMs, the choice of suitable thermally stable electron-transport materials (ETMs) is essential to obtain highly efficient PSCs. Each material constituting the solar cells must have first-rate thermal stability. TiO_2_ itself as an ETM is well-known to be thermally stable up to 500 °C, while standard perovskite is reported to have excellent thermal robustness up to 150 °C [99]. Additionally, light absorbers ought to be exceedingly thermally stable as well. To that end, Seok and Seo developed a new PSC using copper Pc **6** as HTM (Figure 5) [92], and organolead halide perovskite as a light absorber. The authors explored the thermal stability of PSCs prepared from a PbI_2_-enriched mixed perovskite incorporating formaminium(FA)/methylaminium(MA) in the (FAPbI_3_)_0.85_(MAPbBr_3_)_0.15_ ratio. Besides reaching a very high value of average PCE = 17.50% (entry 5, Table 1 and Figure 6a), the PSC showed superior thermal stability. The cell’s stability was compared to other cells, incorporating 2,2′,7,7′-tetrakis(*N*,*N*-di-*p*-methoxyphenylamine)-9,9′-spirobifluorene (spiro-OMeTAD) and poly(triaryl amine) (PTAA). Cells incorporating these HTMs are known to have high PCE values of ~20%; however, their presence reduces the stability of PSCs to thermal stress because of the relatively low glass transition temperature [100,101,102,103,104,105].

Changes in efficiency observed with heat treatments on a hot plate adjusted to several temperatures for 30 min are seen in Figure 6c, where the device incorporating Pc **6** exhibited no observable reduction in the PCE on moving from room temperature to 120 °C and a small decrease at 130 °C. Long-term thermal stability of Pc **6** based PSC, was also tested, and again much higher stability was displayed by this device against the spiro-OMeTAD containing device. Furthermore, even under exposure to ambient air (25 to 30% relative humidity) at 85 °C, the device without encapsulation was stable for 200 h. The device was also stable under thermal cycling tests for 50 cycles at temperatures ranging from −40 to 85 °C in dry air. Notwithstanding the absence of encapsulation, the device practically retained its initial PCE (98% of its initial PCE) [92].

Torres and Nazeeruddin reported a study using Pcs **7** and **8** (Figure 5) as HTMs in PSC devices [93]. The PSCs incorporating Pcs with four peripheral hexylthiophene (**7**) and hexylbisthiophene groups (**8**) were evaluated, and a crucial role of the molecular aggregation of Pcs in HTM layers in the charge transport properties was demonstrated. Using a combination of these Pcs with the mixed-ion modified perovskite light absorber (FAPbI_3_)_0.85_(MAPbBr_3_)_0.15_, PCEs of average 17.1% and 15.50% (entries 6 and 7, Table 1) for devices incorporating Pc **7** and Pc **8**, respectively, were obtained.

Wang and Sun also attempted to modulate the phthalocyanine structure to enhance its solubility in layer formation/deposition. They used Pc **9** (Figure 5) [94], bearing the widely well-known and widely used triphenylamine (TPA) as the electron donor. The other main change was the use of carbon as a cathode layer, a considerably cheaper option than using a noble metal as a counter-electrode [103,104]. Nevertheless, the authors could not improve the state of the art, reaching an average PCE of 13.50% (entry 8, Table 1). The same authors later managed to improve PCE values by using Pc **10** as a HTM (Figure 5) and a MAPbI_3_ perovskite layer as an electron harvester, reaching a PCE = 16.10% (entry 9, Table 1) [95].

Liao, instead of using only TiO_2_ as an electron transport layer (ETL), assembled a bilayer consisting of SnO_2_ and TiO_2_ [96]. The latter is, naturally, the most commonly used ETL [106,107] due to its suitable energy band matching and high transmittance toward visible light. However, the electron recombination rate for this ETL is very high due to low electron mobility, which is always an issue to overcome. Thus, Liao’s research group developed a PSC incorporating copper Pc **10** (Figure 5) as a HTM, carbon as a counter-electrode, a modified perovskite layer as a light absorber with an increased amount of Br ions [Cs_0.05_(MA_0.17_FA_0.83_)_0.95_Pb(I_0.83_Br_0.17_)_3_], and a layered mixture of SnO_2_ and TiO_2_. The device displayed a PCE = 15.39% (entry 9, Table 1) and showed almost no decrease in PCE after 1200 h when stored in ambient air.

Pursuing their intents in improving the ETL capacity, the same authors produced a carbon-based planar heterojunction PSC using high-crystallinity Ni-doped TiO_2_ as the ETL, using copper Pc **10** (Figure 5) as the HTM [97]. They found that 0.01 M Ni doping could cause an increase in the charge mobility of the TiO_2_ film, thus enhancing the charge transport and extraction. An optimized PCE of 17.46% was therefore obtained (entry 10, Table 1), comparable to the best values obtained for Au containing PSCs.

The same authors, who completely replaced TiO_2_ as the ETL and reported a low-temperature processed Zn-doped SnO_2_ (below 200 °C) as the ETL [98], incorporating carbon as counter electrode and Pc **10** (Figure 5) as the HTM, delivered a further step forward in the research on PSCs. They observed improved conductivity for SnO_2_ films doped with 2 mM Zn, resulting in efficient electron transfer and charge recombination suppression. An optimal efficiency of 17.78% was obtained, maintaining almost 100% of their initial efficiencies over 1200 h in ambient air.

### 3.2. Charge Transfer Materials

Transition metal dichalcogenides (TMDs) are layered two-dimensional (2D) materials that attracted much recent interest, mostly due to their capability to modulate materials’ properties by the stacking of different compounds together [108].

This ability is enabled by the weak van der Waals bonding between layers and the absence of hanging bonds at the interface. Since organic molecules are also bonded by van der Waals forces, these readily combine with TMDs, providing synergized heterostructures. Hence, these materials are the most promising semiconductor platforms, with MoS_2_ being the most prominent member [109,110]. As phthalocyanines are robust, chemically stable electron-rich systems with a high capability to generate charge-separated states, these are ideal candidates to produce excellent semiconducting charge transfer materials.

**Figure 7:**
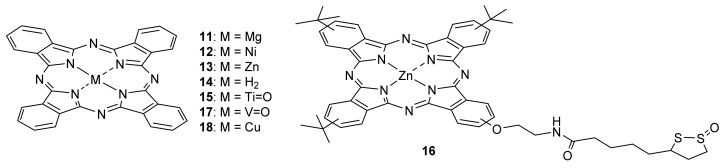
Phthalocyanines for application in semiconducting systems.

For instance, Choi studied the electronic charge transfer between several TMDs, such as MoS_2_, MoSe_2_, and WSe_2_, with Pcs **11** and **12** (Figure 7) [111]. They demonstrated that the photoluminescence emission could be selectively and reversibly modulated through energetically favorable electron transfer from photo-excited TMDs to the metal-Pcs. Nickel Pc **12**, whose reduction potential is lower than the conduction band minima (CBM) of monolayers MoSe_2_ and WSe_2_, but is higher than that of MoS_2_, acts as a photoluminescence quencher of MoSe_2_ and WSe_2_, but not MoS_2_. Similarly, Mg Pc **12** quenches only WSe_2_, as its reduction potential is lower than those of the CBM of WSe_2_, but above those of MoS_2_ and MoSe_2_. The authors also found that photocurrents from TMDs increased more than two-fold, only when the PL was quenched by metalloPcs, further supporting the photoinduced charge transfer mechanism. The quenched PL emission could be fully recovered when the Pc molecules were removed from the TMD surfaces, allowing re-functionalization and recycling.

Other authors studied the influence of zinc Pc **13** (Figure 7) in the interface with MoS_2_ [112,113,114]. Chan found that the optically excited singlet exciton in Pc **13** is able to transfer its electron to MoS_2_ in 80 fs after photoexcitation to form a charge transfer exciton [112]. However, back electron transfer occurs on the time scale of ~1–100 ps, resulting in the formation of a triplet exciton in the Pc layer. The authors attributed this relatively fast singlet–triplet transition to the large singlet–triplet splitting in organic materials and the strong spin–orbit coupling in TMD crystal layers. Furthermore, the same authors demonstrated that the ZnPc **13**/monolyered MoS_2_ behaves similar to typical donor–acceptor interfaces, in which charge transfer excitons dissociate into electron–hole pairs [113]. On the contrary, back electron transfer occurs at ZnPc/bulk-MoS_2_, resulting in the formation of triplet excitons in the zinc Pc, due to the different amount of band bending found in the Zn Pc **13** film deposited on monolayered-MoS_2_ and bulk-MoS_2_.

In addition, Zhai demonstrated the occurrence of ultrafast photoresponse dynamics in monolayered MoS_2_ by using a charge transfer interface based on surface assembled zinc phthalocyanine molecules (Pc **13**, Figure 7) [114]. The formed Pc **13**/MoS_2_ interface led to the separation of photogenerated holes to ZnPc **13** molecules [115], suppressing slow hole trapping in MoS_2_, thus offering the significantly improved photoresponse dynamics in MoS_2_ detectors by almost three orders (from > 20 s to <8 ms for the decay).

Other researchers developed MoS_2_ nanosheets decorated with free base phthalocyanine (Pc **14**, Figure 7) [116]. The semiconducting heterostructure was then studied for its charge transfer properties. Using laterally confined MoS_2_ nanosheets, the conduction band of the semiconductor was positioned between Pc’s S_1_ and S_2_ states, causing bidirectional photoinduced electron transfer processes. On one side, excitation of Pc’s S_2_ state led to electron injection into the MoS_2_ conduction band. However, charge transfer from the dye’s S_1_ transition to the MoS_2_ nanosheet was found to be thermodynamically unfavorable, resulting in intense radiative recombination.

Kummel prepared a van der Waals interface between a monolayer (ML) of titanyl phthalocyanine (Pc **15**, Figure 7) and a ML of MoS_2_ [117]. A strong negative charge transfer from MoS_2_ to TiOPc molecules was observed and the I_ON_/I_OFF_ in backgated MoS_2_ transistors increased by more than two orders of magnitude, while the degradation in the photoluminescence signal was suppressed. The defect passivation methodology used permitted the demonstration that the defect states at low energy levels (near a conduction band edge) was observed with S vacancies, while defects at deep energy levels (in the middle of a band gap) were not observed. Because the Pc **15**/MoS_2_ charge transfer van der Waals interface relied on the nonbonding interaction, it could present limitations in passivating deep-level defect states, which may be overcome by the introduction of a stronger interaction, perhaps a covalent one.

**Figure 8:**
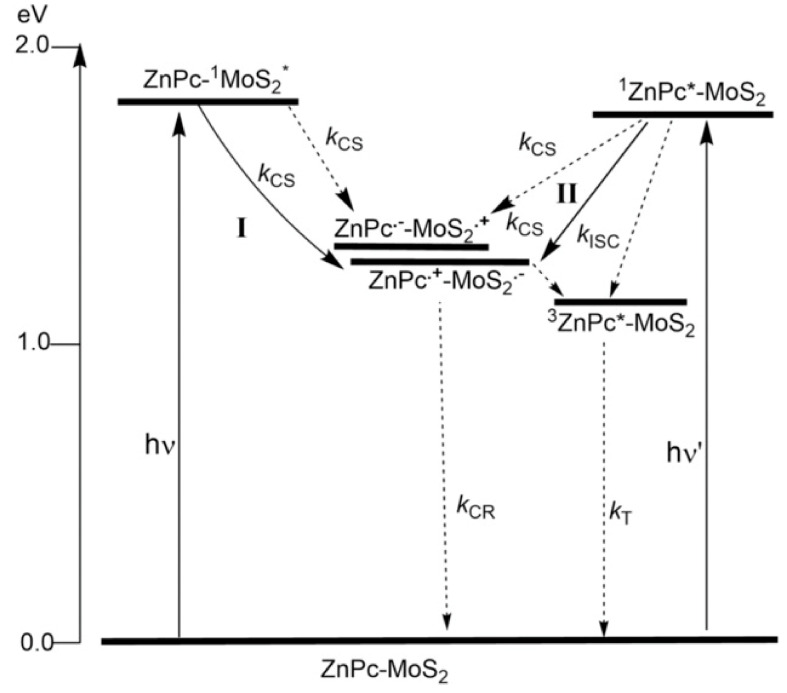
Energy-level diagram showing possible photochemical processes occurring in the Pc **16**-MoS_2_ hybrid material. Solid arrows show major processes; dashed arrows show minor ones. Adapted with permission from ref [118]. Copyright 2019 John Wiley and Sons.

With that in mind, Sastre-Santos, D’Souza, and Tagmatarchis used zinc phthalocyanine Pc **16** (Figure 7) bearing an 1,2-dithiolane oxide linker to functionalize MoS_2_ at defect sites located at the edges of the semiconducting surface [118]. The energy-level diagram depicted in Figure 8 shows different photochemical processes in Pc **16**-MoS_2_, demonstrating a bidirectional electron transfer leading to a charge separated state of Pc **16** (ZnPc•^+^)-MoS_2_•^−^. Marked evidence of the charge transfer in the hybrid material was demonstrated with involvement of excitons generated in MoS_2_ in promoting the charge transfer while the transfer was also possible when Pc **16** was excited, suggesting their potential in light-energy-harvesting devices.

In addition to the charge carriers generated by charge transfer, organic phototransistors are devices that convert incident optical signals to electrical signals with amplification properties, and photomemory devices convert and also store the light information as an electrical signal, which is a building block for optical signal processing and photonic neuromorphic circuits [119]. Sun and Gao prepared organic heterojunction phototransistors based on phthalocyanine/*para*-sexiphenyl (Pc/p-6P) thin films [120,121]. In one case, they used vanadyl Pc **17** (Figure 7) [120]. Under 365 nm ultraviolet light irradiation, the ratio of photocurrent and dark current and photoresponsivity of the phototransistor were about 1.5 × 10^5^ and 87 A/W, respectively. After applying a light pulse (4.2 mW/cm^2^, 100 ms) on the device, the stored current level lasted for ~5000 s with only a 20% decrease, indicating a good photomemory behavior. In the other study, the authors used copper Pc **18** and managed to input an important role of the *para*-sexiphenyl thin film on the performance of CuPc **18**/p-6P heterojunction phototransistors [121]. It acted as a molecular template layer to induce the growth of highly ordered CuPc thin film, improving the charge transport and decreasing the grain boundaries. On the other hand, the p-6P thin film formed an effective heterojunction with Pc **18** thin film, enhancing the light absorption and photogenerated carriers. Under 365 nm ultraviolet light irradiation, the ratio of photocurrent to dark current and photoresponsivity of Pc **18**/p-6P heterojunction phototransistors reached ca. 2.2 × 10^4^ and 4.3 × 10^2^ A/W, respectively, which are much larger than that of Pc **18** phototransistors of ca. 2.7 × 10^2^ and 7.3 A/W, respectively.

### 3.3. Photocatalysis

The use of phthalocyanines in photocatalysis is useful for light-triggered processes. Pcs are particularly applicable in composited photocatalytic materials that need visible light, deliberately inspired from photodynamic therapy in medical applications related to cancer treatment, or through the coupling of photosensitizing Pcs with semiconductor materials, intentionally inspired from cells in photovoltaic applications.

**Figure 9:**
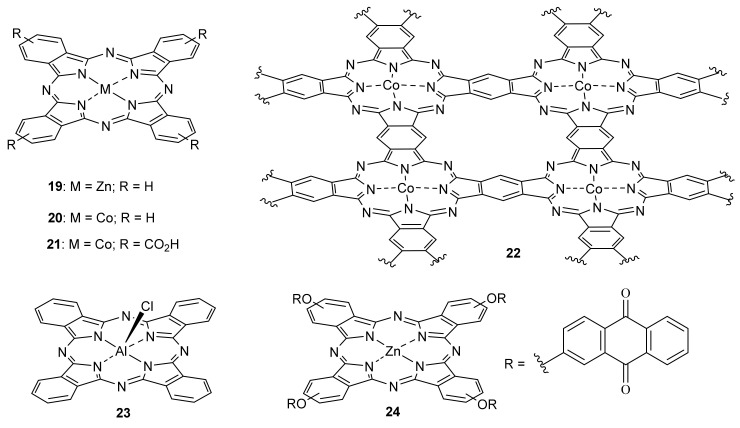
Pcs used in photocatalysis.

For instance, Tang and Jing reported the synthesis of an H-bond linked unsubstituted zinc phthalocyanine Pc **19**/BiVO_4_ nanosheet composite (Figure 9) [122], which managed to function as a visible-light-driven photocatalyst for converting CO_2_ into CO and CH_4_ (under monochromatic beams at 520 and 660 nm), with the Pc presence as cause for a 16-fold increase over the BIVO4 performance (only at 520 nm).

On the other hand, Jain prepared a nanoparticulated composite by the grafting of cobalt (II) Pc **19** (Figure 9) on the core-shell Ni/NiO semiconductor for the photocatalytic reduction of CO_2_ to methanol under visible illumination [123]. The photocatalyst (10% Pc **19**) exhibited a remarkable enhancement in the yield of methanol as compared to the semiconductor Ni/NiO and Pc **19** for CO_2_ reduction under identical conditions (a 12- and 8.5-fold increase, respectively). The maximum yield of methanol was found to be a ~3640 μmol/g catalyst with the conversion rate of ~152 μmol/g h, with reusability up to four times without loss of activity. The same group further reported another photocatalyst [124], which was prepared by intercalation of tetracarboxy-substituted Pc **20** (Figure 9) into graphitic carbon nitride g-C_3_N_4_. The best results were obtained with the photocatalyst containing 10% Pc **20**, with an 11- and a 7-fold increase in methanol production when compared to g-C_3_N_4_ and Pc **20** alone, respectively. The maximum methanol yields were 12,930 μmol/g catalyst after 24 h irradiation (rate = 540 μmol/g h).

Reisner prepared a photocatalyst based on mesoporous graphitic carbon nitride (mpg-CNx), incorporating the polymeric Co(II) Pc **21** (Figure 9) [125]. Under full solar spectrum irradiation, the photocatalyst mpg-CNx:Pc **22** (~12 mmol Co/g catalyst) generated a 1000 mmol CO/g catalyst after 48 h with 85% selectivity (TON_CO_ = 85), a 65% increase in activity compared to that under visible light alone (607 mmol CO/g after 48 h, TON_CO_ = 51). However, the catalyst only retained 85% of its activity after the second run.

Boyer used Pc **23** (Figure 9) in the radical polymerization of (meth)acrylate and (meth)acrylamide monomers [126], in combination with the solvent *N*-methyl-2-pyrrolidone (NMP), as a photoinitiation catalyst under visible and near-infrared light. The Pc reacted via a reductive quenching pathway to oxidize NMP to a NMP radical, which could directly initiate the polymerization of monomers. (Meth)acrylate and (meth)acrylamide monomers were initiated through a photoinduced electron/energy transfer process and subsequently controlled through reversible addition–fragmentation chain transfer equilibria. Thanks to the high molar extinction coefficients of Pc **23**, polymerization was accessible with an ultralow (5 ppm) catalyst concentration, besides tolerance to elemental oxygen.

Yilmaz and Versace reported Pc **24** (Figure 9) as a photoinitiator for free-radical and cationic photopolymerization reactions [127]. They combined the visible light-sensitive anthraquinone functionality with the phthalocyanine, further using a suitable electron acceptor for cationic polymerization (iodonium salt), and an electron donor (methyldiethanol amine, MDEA), or H-donor (thiol derivative, trithiol), for the free-radical polymerization. These combinations, carried out under visible light irradiation, promoted high acrylate and epoxy conversions with efficiency comparable to well-known camphorquinone-based photoinitiating systems [128,129].

**Figure 10:**
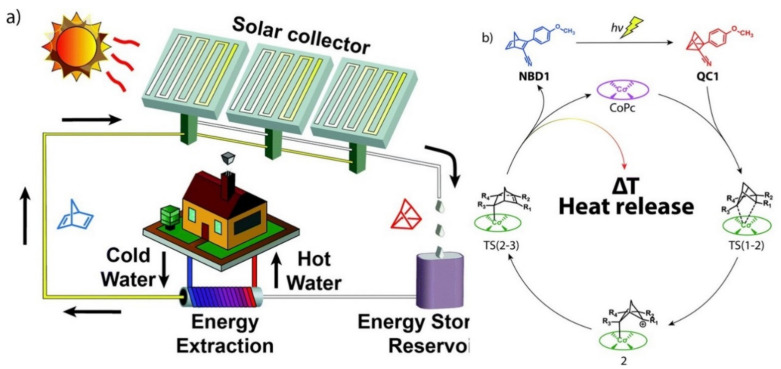
(**a**) Operating set up based on the MOST concept; (**b**) Catalytic cycle for the back-reaction. Adapted with permission from ref [130]. Copyright 2019 The Royal Society of Chemistry.

Moth-Poulsen used Pc **20** (Figure 9) as a photocatalyst in a molecular photoswitching system [130], so-called molecular solar thermal energy storage (MOST). In this interesting concept, sunlight is collected and stored via photochemical reaction under flow conditions. When energy is required, a solution of the metastable molecule can be passed through a catalytic bed reactor to release the energy in the form of heat, which could be used for, in this instance, heating water or creating steam (Figure 10a). In the study, the authors used a norbornadiene derivative (**NBD1**), with improved absorptivity (λ_max_ = 326 nm = 1.3 × 10^4^ M^−1^ cm^−1^. Pc **20** was then used to promote the photoisomerization of **NBD1** into quadricyclane **QC1**, the couple isomer of the former compound, with high photoisomerization quantum yield (61%). **QC1** was characterized by its long half-life (t_1/2_ = 30 days at 25 °C) and high solubility (C_max_ = 1.52 M for **QC1** in toluene). Moreover, by using the small reaction center of the heterogeneous Pc **20** photocatalyst (Figure 10b), the stored energy could be efficiently released. A macroscopic heat release of up to ~63 °C using a 1.5 M solution of **QC1** was measured, with sustaining a reaction rate in toluene of 1.2 × 10^4^ s^−1^ M^−1^ and a minimum TON of 482.

Degradation of pollutants present in the environment is crucial ensure continuity of life on Earth, since avoiding its pollution has been an impossible design. Among the many removal/degradation processes, advanced oxidation processes (AOPs) refer specifically to processes in which the oxidation of compounds occurs primarily through reactions with reactive oxygen species (ROS), and the reaction of these species with the target pollutants in water [67,69,131]. AOPs are considered as effective water treatment technologies. Particularly, semiconductor photocatalysis is one of the most effective technologies for the destruction of pollutants in water. As already pointed out, Pcs are photosensitizers that efficiently generate ROS and, hence, have been used in the photocatalytic degradation of many pollutants.

**Figure 11:**
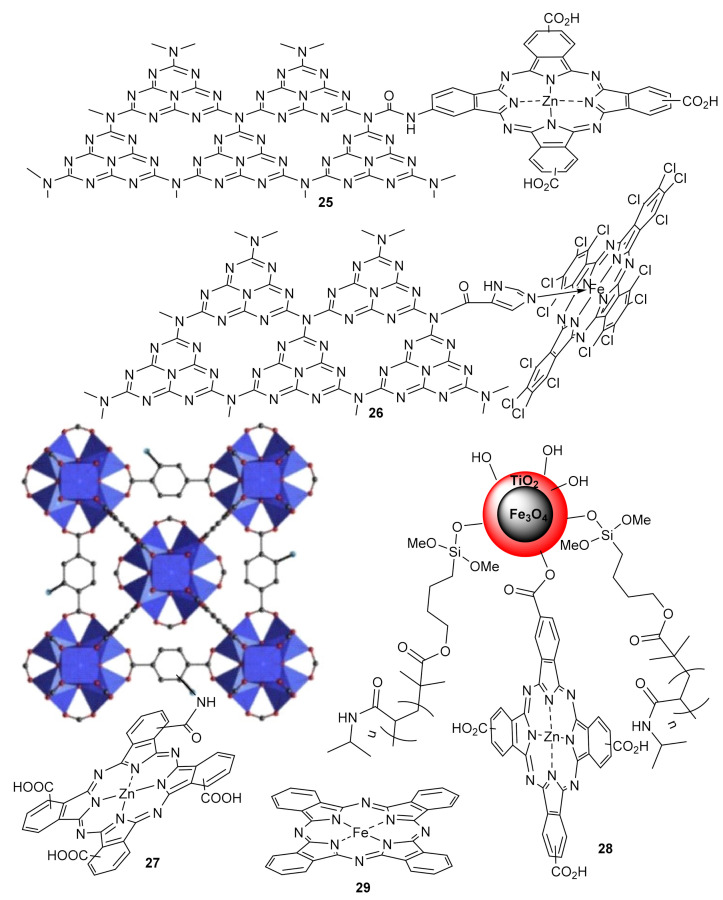
Immobilized phthalocyanine photocatalysts for degradation of pollutants. Structure of compound **27** was adapted with permission from ref [136]. Copyright 2017 Elsevier.

For instance, Lu’s group reported the synthesis of photocatalysts Pc **25** and **26** (Figure 11) for the efficient and reusable photodegradation of pollutants rhodamine B (RhB) [132,133,134] and 4-chlorophenol (4-CP) [132,133] carbamazepine (CBZ) [133,134,135] and sulfaquinoxaline sodium (SQS) [134] under visible irradiation. In all cases, besides photogenerated holes, the presence of singlet oxygen (^1^O_2_), superoxide radical (•O_2_^−^), and hydroxyl radical (•OH) was evidenced in the visible light-responsive catalytic system.

Liang and Li prepared photocatalyst **27** (Figure 11) [136], consisting of a zirconium-based metal organic framework [MOF UIO-66 (NH_2_)], covalently coupled with α-tetracarboxy substituted Zn Pc, Which was prepared via a facile condensation process. Compared with the mixture of α-tetracarboxy Zn Pc and Zr-UIO-66 by impregnation, **27** presented an enhanced photocatalytic activity for the degradation of methylene blue (MB) under visible-light irradiation. The formation of strong covalent bonds and a synergistic interaction between α-tetracarboxy Zn Pc and UIO-66 (NH_2_) proved more effective, retaining its stability after five recycles.

Duan promoted the covalent immobilization of β-tetracarboxy Zn Pc on the surface of TiO_2_ coated magnetite, obtaining catalyst **28** (Figure 11) [137]. The absorption light range was effectively broadened from the ultraviolet to visible region, and photocatalytic studies revealed a high decomposition rate of RhB under visible and sun light, retaining its photocatalytic activity for four cycles.

Dong produced a visible-light-driven water-fueled micromotor based on iron Pc **29** (Figure 11) [138]. These micromotors are micrometer-sized machines, capable to undergo photoinduced self-propelling movement. In this case, the photocatalyst, prepared by emulsification of a gelatin solution of Pc **29** in olive oil, yielding the spherical micromotors, exhibited visible-light-driven self-propulsion behavior using water fuel based on the photocatalytic reaction and self-diffusiophoresis mechanism. The system showed good photocatalytic activity in the degradation of the RhB pollutant, with a normalized reaction rate constant of 2.49 × 10^−2^ L/m^2^ s.

We can generally observe that Pc’s structure is not definite in providing adequate properties to any certain photocatalytic purpose. The most important issue regards the “to all cost” leaching prevention, since it hampers reusability to a large extent.

### 3.4. Nonlinear Optics

The control of the transmission of the energy transported by optical waves is of extreme importance for the realization of advanced technologies that require high speed of operation and fast switching. Nonlinear optics (NLO) are an example of such a technological application, where phthalocyanines may play a prominent role, motivated by the fact that the optical properties of such annulated systems can be finely modulated in a controlled fashion by changing the chemical structure of the complex. These changes involve the variation of the central metal, the extent of electronic conjugation of the ring, the nature and the number of peripheral ligands, and the eventual introduction of axial ligands coordinated by central metals with a valence higher than +2 [64,65,139,140]. The definition of NLO mechanisms can be seen in references [64,65].

**Figure 12:**
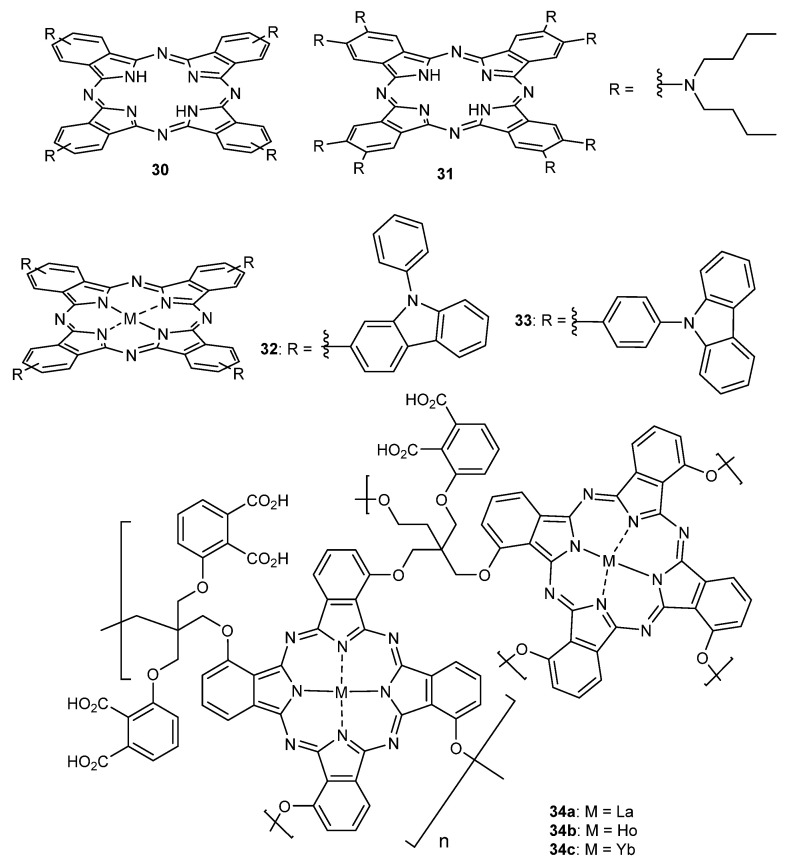
Phthalocyanines for nonlinear optics applications.

For instance, Qi and Jiang reported Pcs **30** and **31** (Figure 12), bearing four and eight dibutylamino groups, respectively [141]. Electronic absorption spectroscopic studies revealed the more effective conjugation of the nitrogen lone pair of electrons in the dibutylamino side chains with the Pc π system in **30** than in **31**, which, in turn, resulted in superior third-order NLO properties. This was demonstrated by a larger effective imaginary third-order molecular susceptibility, a crucial parameter to define nonlinearity of a material ((Im{χ^(3)^}) of 6.5 × 10^−11^ esu for the former and 3.4 × 10^−11^ esu for the latter one).

**Figure 13:**
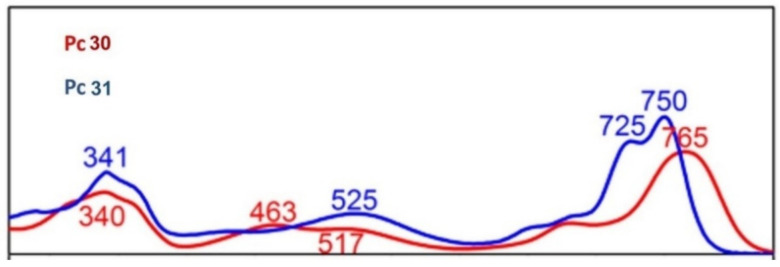
Comparison in the electronic absorption spectra between Pcs **30** and **31**, recorded in CHCl_3_. Adapted with permission from ref [141]. Copyright 2016 American Chemical Society.

The electronic absorption spectra of Pcs **30** and **31** were recorded in CHCl_3_, where **30** shows a characteristic non-aggregated absorption spectrum of the metal-free phthalocyanines with a relatively broadened Soret absorption band appearing at 340 nm and a broad Q band at 765 nm. Of note, theoretical calculations revealed that, unlike typical metal-free Pcs [142,143], the Q band for **30** did not get split because of the more effective p-π conjugation between the peripheral nitrogen atoms and the central phthalocyanine chromophore, therefore extending the effective conjugation system for Pc **30** (Figure 13).

The nonlinear optical properties of carbazole substituted phthalocyanines were also reported by Giribabu and Rao Soma, having the carbazole units linked either through aromatic C-C bonds or C-N bonds (Figure 13, **32** and **33**, respectively) [144]. This particularity was revealed as crucial for the NLO properties of **32** and **33**, which were investigated using the Z-scan technique and femtosecond (fs) pulses, with kHz and MHz repetition rates, where two-photon absorption (TPA) was the dominant mechanism observed. At 800 nm under kHz pulses, Pc **32** displayed (Im{χ^(3)^}) = 3.9 × 10^−15^ esu while Pc **33** showed a (Im{χ^(3)^}) = 7.9 × 10^−14^ esu, 20 times higher.

The nonlinear optical properties of the polymeric carboxyl phthalocyanines with lanthanum (**34a**), holmium (**34b**), and ytterbium (**34c**) as central metals (Figure 12) were investigated using a by-scan method using a picosecond 532 nm laser [145]. NLO response was attributed to the reverse saturable absorption and self-focus refraction, decreasing in the order **34a** > **34b** > **34c** with Im{χ^(3)^} values of 4.86 × 10^−12^ esu, 4.18 × 10^−12^ esu and 3.60 × 10^−12^ esu, respectively. Furthermore, **34a** showed a limiting threshold (that is, the incident fluence at which the transmittance drops to half) of 0.32 J/cm^2^, while **34b** and **34c** displayed higher values of 0.5 J/cm^2^ and 0.8 J/cm^2^, respectively.

**Figure 14:**
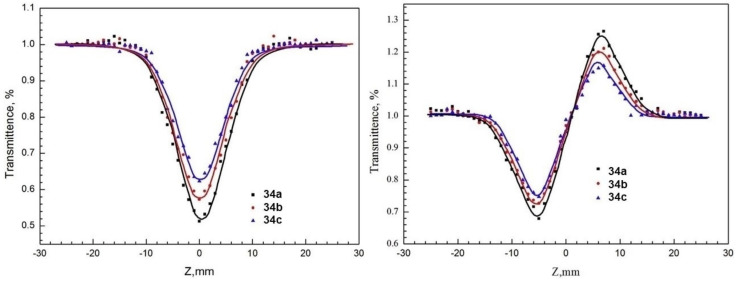
The nonlinear absorption and fitting curves (solid lines) of polymeric carboxyl phthalocyanines Z-scan (left: closed aperture; right: open aperture). Adapted with permission from ref [144]. Copyright 2017 American Chemical Society.

Figure 14 shows the nonlinear absorption of the three polymeric carboxyl phthalocyanines under open aperture conditions in the Z-scan test (left). All curves exhibit valleys, which indicate the reverse saturable absorption with a positive coefficient. Under closed aperture conditions (right), the valley-to-peak forms suggest a self-focus effect of nonlinear reflection [145].

**Figure 15:**
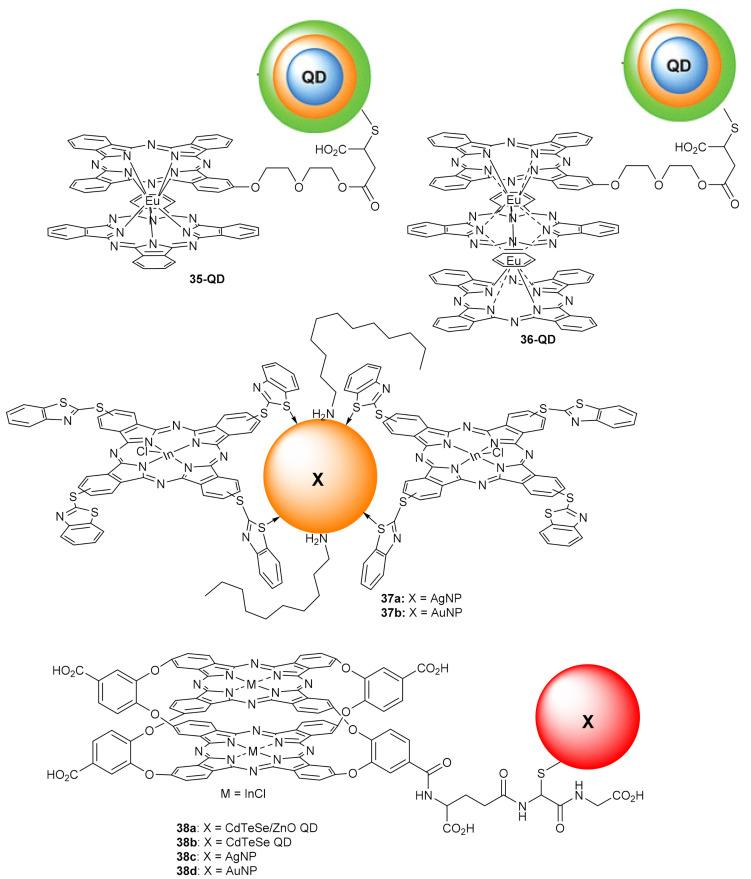
Phthalocyanine hybrid materials for NLO.

**Figure 16:**
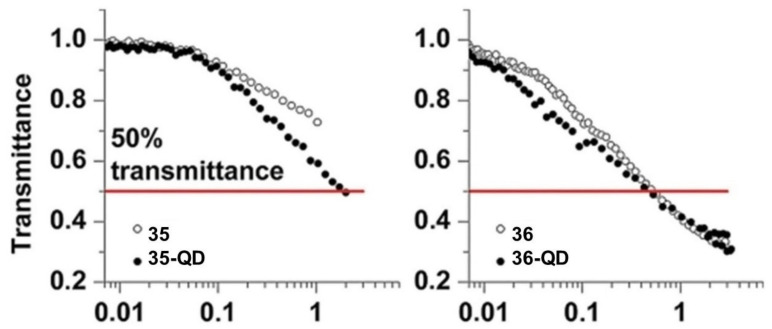
Transmittance of sandwich complexes and their conjugates with QDs depending on the incident fluence, performed at a pulse rate of 10 ns and I_0_ ≈ 0.33 GW/cm^2^. The red solid line corresponds to 50% transmittance (limiting threshold). Adapted with permission from ref [146]. Copyright 2017 John Wiley and Sons.

Prolifically, Nyokong’s group reported the NLO properties of several phthalocyanine-based hybrid materials, of which we highlight compound **35**–**38** (Figure 15) [146,147,148]. For instance, double- and triple-decker phthalocyanines **35-QD** and **36-QD** were linked to mercaptosuccinic acid-capped ternary CdSeTe/CdTeS/ZnSeS quantum dots (QD), and their optical power limiting (OPL) properties were evaluated by the open-aperture Z-scan technique (532 nm laser and pulse rate of 10 ns) [146]. It was observed that both lowering of molecular symmetry and expansion of the π electron system upon moving from double- to triple-decker complexes significantly improves the OPL characteristics, making the low-symmetry triple-decker complex **36-QD** the most efficient optical limiter, affording 50% lowering of light transmittance below 0.5 J/cm^2^ input fluence, with a Im{χ^(3)^} value of 4.2 × 10^−11^ esu (Figure 16).

The same authors measured the nonlinear absorption behavior of hybrids **36a**–**b** by using the open aperture z-scan technique with excitation pulses of 10 ns at a wavelength of 532 nm and a peak intensity of 50.0 MW/cm^2^ [147]. A comparison with similar hybrid materials continuing other central moieties demonstrated that the In(III) containing complexes showed the best OPL performance, limiting thresholds of 0.14 and 0.12 for **36a** and **36b**, respectively. In sequence, the same group covalently attached a ball-type indium phthalocyanine to glutathione capped (Ag, Au, CdTeSe, CdTeSe/ZnO) nanoparticles [148]. The optical limiting threshold ranged from 0.40 to 0.78 J/cm^2^, with the complex **37a** accounting for the most improved triplet state parameters and nonlinear optical behavior (0.40 J/cm^2^).

## 4. Conclusions

Phthalocyanines find many photomaterial applications, where their photo-properties need to reach exquisite levels; the objectives are to modulate Pcs’ photophysical properties, while ensuring their maneuverability in adequate media and ensuring stability for the macrocycle. Since Pcs usually act in combination with other materials, careful interfacial engineering should hold the key to enhance the applicability of phthalocyanine as photomaterials.

Many authors have been successful in their endeavors in recent years. Most of the applications require the utilization of previous knowledge, particularly regarding strategies to enhance Pcs’ photophysical characteristics; nevertheless, a great deal of discoveries and milestones arise from empirical knowledge.

## Figures and Tables

**Table 1:** Photovoltaic parameters obtained for perovskite solar cells employing phthalocyanines as hole-transporting materials.

Entry	J_SC_ (mA/cm^2^)	V_OC_ (V)	FF	PCE (%)	Cell Configuration ^1^	Ref.
1	11.79	1.04	0.64	8.24	dTiO_2_/mpTiO_2_/perovskite/Pc **4a**/Au	[90]
2	20.83	1.07	0.64	14.35	dTiO_2_/mpTiO_2_/perovskite/Pc **4b**/Au	[89]
3	20.46	1.06	0.58	12.72	dTiO_2_/mpTiO_2_/perovskite/Pc **4c**/Au	[89]
4	21.00	1.10	0.68	15.74	dTiO_2_/mpTiO_2_/perovskite/Pc **3 4**/Au	[91]
5	22.60	1.05	0.73	17.50	mpTiO_2_/m-perovskite/Pc **6**/Au	[92]
6	20.28	1.05	0.80	17.10	mpTiO_2_/m-perovskite/Pc **7**/Au	[93]
7	20.16	1.10	0.69	15.50	mpTiO_2_/m-perovskite/Pc **8**/Au	[93]
8	21.00	0.98	0.63	13.5	mpTiO_2_/m-perovskite/Pc **9**/Carbon	[94]
9	20.80	1.05	0.74	16.10	dTiO_2_/mpTiO_2_/MAPbI_3_-perovskite/Pc **10**/Carbon	[95]
10	23.28	0.98	0.67	15.39	SnO_2_:TiO_2_/m-perovskite/Pc **10**/Carbon ^2^	[96]
11	22.41	1.07	0.73	17.46	Ni:TiO_2_/m-perovskite/Pc **10**/Carbon ^2^	[97]
12	23.40	1.10	0.69	17.78	FTO/Zn:SnO_2_/m-perovskite/Pc **10**/Carbon ^2,3^	[98]

Performances were recorded under full Sun AM 1.5G light (solar simulator 100 mW/cm^2^). J_SC_ = short-circuit current density; V_OC_ = open-circuit voltage; FF = fill factor; PCE = power conversion efficiency; dTiO_2_ = dense TiO_2_; mpTiO_2_ = mesoporous TiO_2_; m-perovskite = modified mixed-ion perovskite with ratio (FAPbI_3_)_0.85_(MAPbBr_3_)_0.15_; ^1^ all cells incorporate fluorine-doped tin oxide (FTO) as lower layer (not represented); ^2^ modified perovskite as Cs_0.05_(MA_0.17_FA_0.83_)_0.95_Pb(I_0.83_Br_0.17_)_3_; ^3^ this cell is TiO_2_ free.

## Supplementary Materials

The following are available online at, Figure S1: Results obtained using search strings (phthalocyanin*) and (phthalocyanin* AND photo*) present in the title and/or abstract and/or keywords in search engines Scopus and Web of Science (WoS), Figure S2: Country weight in publication of articles by five-year span, from 1971 to 2020. Articles were found by using search string “phthalocyanin*” followed by refining with term “photo*”. (a) 1971–1975; (b) 1976–1980; (c) 1981–1985; (d) 1986–1990; (e) 1991–1995; (f) 1996–2000; (g) 2001–2005; (h) 2006–2010; (i) 2011–2015; (j) 2016–2020.

## References

[1] Dini D., Hanack M., Kadish K.M., Guilard R., Smith K.M. (2003). Physical Properties of Phthalocyanine-based Materials. The Porphyrin Handbook, Phthalocyanines: Properties and Materials.

[2] L’Her M., Pondaven A., Kadish K.M., Guilard R., Smith K.M. (2003). Electrochemistry of Phthalocyanines. The Porphyrin Handbook, Phthalocyanines: Spectroscopic and Electrochemical Characterization.

[3] Nyokong T., Antunes E., Kadish K.M., Smith K.M., Guilard R. (2012). Photochemical and Photophysical Properties of Metallophthalocyanines. Handbook of Porphyrin Science, with Applications to Chemistry, Physics, Materials Science, Engineering, Biology and Medicine.

[4] Fukuda T., Kobayashi N., Kadish K.M., Smith K.M., Guilard R. (2012). UV-Visible Absorption Spectroscopic Properties of Phthalocyanines and Related Macrocycles. Handbook of Porphyrin Science, with Applications to Chemistry, Physics, Materials Science, Engineering, Biology and Medicine.

[5] Calvete M.J.F. (2016). Phthalocyanines as top materials. Future Trends for Top Materials.

[6] Nemykin V.N., Lukyanets E.A., Kadish K.M., Smith K.M., Guilard R. (2010). The Key Role of Peripheral Substituents in the Chemistry of Phthalocyanines. Handbook of Porphyrin Science with Applications to Chemistry, Physics, Materials Science, Engineering, Biology and Medicine: Synthetic Methodology.

[7] Darwent J.R., Douglas P., Harriman A., Porter G., Richoux M.C. (1982). Metal Phthalocyanines and Porphyrins as Photosensitizers for Reduction of Water to Hydrogen. Coord. Chem. Rev..

[8] Sorokin A.B. (2013). Phthalocyanine Metal Complexes in Catalysis. Chem. Rev..

[9] Calvete M.J.F., Silva M., Pereira M.M., Burrows H.D. (2013). Inorganic helping organic: Recent advances in catalytic heterogeneous oxidations by immobilised tetrapyrrolic macrocycles in micro and mesoporous supports. RSC Adv..

[10] Carrion E.N., Loas A., Patel H.H., Pelmus M., Ramji K., Gorun S.M. (2018). Fluoroalkyl phthalocyanines: Bioinspired catalytic materials. J. Porphyr. Phthalocyanine.

[11] Zhou R., Josse F., Gopel W., Ozturk Z.Z., Bekaroglu O. (1996). Phthalocyanines as sensitive materials for chemical sensors. Appl. Organomet. Chem..

[12] Kumar A., Meunier-Prest R., Bouvet M. (2020). Organic Heterojunction Devices Based on Phthalocyanines: A New Approach to Gas Chemosensing. Sensors.

[13] Stefanov C., van Staden J.F., Stefan-van Staden R.I. (2020). Review-Enzymatic and Non-Enzymatic (bio)sensors Based on Phthalocyanines. A Minireview. ECS J. Solid State Sci. Technol..

[14] Cook M.J. (1996). Thin film formulations of substituted phthalocyanines. J. Mater. Chem..

[15] Papageorgiou N., Salomon E., Angot T., Layet J.M., Giovanelli L., Le Lay G. (2004). Physics of ultra-thin phthalocyanine films on semiconductors. Prog. Surf. Sci..

[16] Valli L. (2005). Phthalocyanine-based Langmuir-Blodgett films as chemical sensors. Adv. Colloid Interface Sci..

[17] Melville O.A., Lessard B.H., Bender T.P. (2015). Phthalocyanine-Based Organic Thin-Film Transistors: A Review of Recent Advances. ACS Appl. Mater. Interfaces.

[18] Giroudgodquin A.M., Maitlis P.M. (1991). Metallomesogens—Metal-Complexes in Organized Fluid Phases. Angew. Chem. Int. Ed..

[19] Laschat S., Baro A., Steinke N., Giesselmann F., Hagele C., Scalia G., Judele R., Kapatsina E., Sauer S., Schreivogel A. (2007). Discotic liquid crystals: From tailor-made synthesis to plastic electronics. Angew. Chem. Int. Ed..

[20] Basova T., Hassan A., Durmus M., Gurek A.G., Ahsen V. (2016). Liquid crystalline metal phthalocyanines: Structural organization on the substrate surface. Coord. Chem. Rev..

[21] Hanack M., Lang M. (1994). Conducting Stacked Metallophthalocyanines and Related-Compounds. Adv. Mater..

[22] Inabe T., Tajima H. (2004). Phthalocyanines—Versatile components of molecular conductors. Chem. Rev..

[23] Gsanger M., Bialas D., Huang L.Z., Stolte M., Wurthner F. (2016). Organic Semiconductors based on Dyes and Color Pigments. Adv. Mater..

[24] Ozdemir P.S., Ozguney A.T. (2017). Chemical structures of phthalocyanine based dyestuffs and their useage in production of functional textiles. PAJES.

[25] Benkhaya S., M’Rabet S., El Harfi A. (2020). A review on classifications, recent synthesis and applications of textile dyes. Inorg. Chem. Commun..

[26] Imahori H., Umeyama T., Ito S. (2009). Large pi-Aromatic Molecules as Potential Sensitizers for Highly Efficient Dye-Sensitized Solar Cells. Acc. Chem. Res..

[27] Bottari G., de la Torre G., Guldi D.M., Torres T. (2010). Covalent and Noncovalent Phthalocyanine-Carbon Nanostructure Systems: Synthesis, Photoinduced Electron Transfer, and Application to Molecular Photovoltaics. Chem. Rev..

[28] Li X.Y., Wang H.X., Wu H.X. (2010). Phthalocyanines and Their Analogs Applied in Dye-Sensitized Solar Cell. Functional Phthalocyanine Molecular Materials.

[29] Walter M.G., Rudine A.B., Wamser C.C. (2010). Porphyrins and phthalocyanines in solar photovoltaic cells. J. Porphyr. Phthalocyanine.

[30] Hanack M., Schneider T., Barthel M., Shirk J.S., Flom S.R., Pong R.G.S. (2001). Indium phthalocyanines and naphthalocyanines for optical limiting. Coord. Chem. Rev..

[31] de la Torre G., Vaquez P., Agullo-Lopez F., Torres T. (2004). Role of structural factors in the nonlinear optical properties of phthalocyanines and related compounds. Chem. Rev..

[32] Calvete M., Yang G.Y., Hanack M. (2004). Porphyrins and phthalocyanines as materials for optical limiting. Synth. Met..

[33] Pereira A., Soares A.R.M., Calvete M.J.F., de la Torre G. (2009). Recent developments in the synthesis of homo- and heteroarrays of porphyrins and phthalocyanines. J. Porphyr. Phthalocyanine.

[34] Calvete M.J.F. (2012). Near-infrared absorbing organic materials with nonlinear transmission properties. Int. Rev. Phys. Chem..

[35] Drobizhev M., Makarov N.S., Rebane A., de la Torre G., Torres T. (2008). Strong two-photon absorption in push-pull phthalocyanines: Role of resonance enhancement and permanent dipole moment change upon excitation. J. Phys. Chem. C.

[36] Drobizhev M., Makarov N.S., Stepanenko Y., Rebane A. (2006). Near-infrared two-photon absorption in phthalocyanines: Enhancement of lowest gerade-gerade transition by symmetrical electron-accepting substitution. J. Chem. Phys..

[37] Marin M.L., Santos-Juanes L., Arques A., Amat A.M., Miranda M.A. (2012). Organic Photocatalysts for the Oxidation of Pollutants and Model Compounds. Chem. Rev..

[38] Das S., Daud W. (2014). A review on advances in photocatalysts towards CO2 conversion. RSC Adv..

[39] Bonnett R. (1995). Photosensitizers of the Porphyrin and Phthalocyanine Series for Photodynamic Therapy. Chem. Soc. Rev..

[40] Hamblin M.R., Hasan T. (2004). Photodynamic therapy: A new antimicrobial approach to infectious disease?. Photochem. Photobiol. Sci..

[41] Jori G., Fabris C., Soncin M., Ferro S., Coppellotti O., Dei D., Fantetti L., Chiti G., Roncucci G. (2006). Photodynamic therapy in the treatment of microbial infections: Basic principles and perspective applications. Lasers Surg. Med..

[42] Maisch T. (2009). A New Strategy to Destroy Antibiotic Resistant Microorganisms: Antimicrobial Photodynamic Treatment. Mini-Rev. Med. Chem..

[43] Lovell J.F., Liu T.W.B., Chen J., Zheng G. (2010). Activatable Photosensitizers for Imaging and Therapy. Chem. Rev..

[44] Sekkat N., van den Bergh H., Nyokong T., Lange N. (2012). Like a Bolt from the Blue: Phthalocyanines in Biomedical Optics. Molecules.

[45] Josefsen L.B., Boyle R.W. (2012). Unique Diagnostic and Therapeutic Roles of Porphyrins and Phthalocyanines in Photodynamic Therapy, Imaging and Theranostics. Theranostics.

[46] Calvete M.J.F., Simoes A.V.C., Henriques C.A., Pinto S.M.A., Pereira M.M. (2014). Tetrapyrrolic Macrocycles: Potentialities in Medical Imaging Technologies. Curr. Org. Synth..

[47] Braun A., Tcherniac J. (1907). The products of the action of acetanhydride on phthalamide. Ber. Dtsch. Chem. Ges..

[48] de Diesbach H., von der Weid E. (1927). Some salt complexes of o-dinitriles with copper and pyridine. Helv. Chim. Acta.

[49] Linstead R.P. (1934). Phthalocyanines part I A new type of synthetic colouring matters. J. Chem. Soc..

[50] Abrahamse H., Hamblin M.R. (2016). New photosensitizers for photodynamic therapy. Biochem. J..

[51] Li X., Lee S., Yoon J. (2018). Supramolecular photosensitizers rejuvenate photodynamic therapy. Chem. Soc. Rev..

[52] Roguin L.P., Chiarante N., Vior M.C.G., Marino J. (2019). Zinc(II) phthalocyanines as photosensitizers for antitumor photodynamic therapy. Int. J. Biochem. Cell Biol..

[53] Li X., Zheng B.D., Peng X.H., Li S.Z., Ying J.W., Zhao Y.Y., Huang J.D., Yoon J. (2019). Phthalocyanines as medicinal photosensitizers: Developments in the last five years. Coord. Chem. Rev..

[54] Lo P.C., Rodriguez-Morgade M.S., Pandey R.K., Ng D.K.P., Torres T., Dumoulin F. (2020). The Unique Features and Promises of Phthalocyanines as Advanced Photosensitisers for Photodynamic Therapy of Cancer. Chem. Soc. Rev..

[55] Zheng B.D., He Q.X., Li X.S., Yoon J., Huang J.D. (2021). Phthalocyanines as contrast agents for photothermal therapy. Coord. Chem. Rev..

[56] Zhang Y.M., Lovell J.F. (2017). Recent applications of phthalocyanines and naphthalocyanines for imaging and therapy. WIREs Nanomed. Nanobiotechnol..

[57] Calvete M.J.F., Pinto S.M.A., Pereira M.M., Geraldes C. (2017). Metal coordinated pyrrole-based macrocycles as contrast agents for magnetic resonance imaging technologies: Synthesis and applications. Coord. Chem. Rev..

[58] Calvete M.J.F., Pinto S.M. (2017). Synthesis of Pyrrole-Based Macrocycles as Molecular Probes for Multimodal Imaging Techniques: Recent Trends. Curr. Org. Synth..

[59] Merkes J.M., Zhu L.M., Bahukhandi S.B., Rueping M., Kiessling F., Banala S. (2020). Photoacoustic Imaging Probes Based on Tetrapyrroles and Related Compounds. Int. J. Mol. Sci..

[60] Dabrowski J.M., Pucelik B., Regiel-Futyra A., Brindell M., Mazuryk O., Kyziol A., Stochel G., Macyk W., Arnaut L.G. (2016). Engineering of relevant photodynamic processes through structural modifications of metallotetrapyrrolic photosensitizers. Coord. Chem. Rev..

[61] de la Torre G., Bottari G., Torres T. (2017). Phthalocyanines and Subphthalocyanines: Perfect Partners for Fullerenes and Carbon Nanotubes in Molecular Photovoltaics. Adv. Energy Mater..

[62] Brogdon P., Cheema H., Delcamp J.H. (2018). Near-Infrared-Absorbing Metal-Free Organic, Porphyrin, and Phthalocyanine Sensitizers for Panchromatic Dye-Sensitized Solar Cells. ChemSusChem.

[63] Urbani M., de la Torre G., Nazeeruddin M.K., Torres T. (2019). Phthalocyanines and porphyrinoid analogues as hole- and electron-transporting materials for perovskite solar cells. Chem. Soc. Rev..

[64] Dini D., Calvete M.J.F., Hanack M. (2016). Nonlinear Optical Materials for the Smart Filtering of Optical Radiation. Chem. Rev..

[65] Calvete M.J.F., Dini D. (2018). Conjugated macrocyclic materials with photoactivated optical absorption for the control of energy transmission delivered by pulsed radiations. J. Photochem. Photobiol. C.

[66] Gounden D., Nombona N., van Zyl W.E. (2020). Recent advances in phthalocyanines for chemical sensor, non-linear optics (NLO) and energy storage applications. Coord. Chem. Rev..

[67] Fernandez L., Esteves V.I., Cunha A., Schneider R.J., Tome J.P.C. (2016). Photodegradation of organic pollutants in water by immobilized porphyrins and phthalocyanines. J. Porphyr. Phthalocyanine.

[68] Cecconi B., Manfredi N., Montini T., Fornasiero P., Abbotto A. (2016). Dye-Sensitized Solar Hydrogen Production: The Emerging Role of Metal-Free Organic Sensitizers. Eur. J. Org. Chem..

[69] Youssef Z., Colombeau L., Yesmurzayeva N., Baros F., Vanderesse R., Hamieh T., Toufaily J., Frochot C., Roques-Carmes T., Acherar S. (2018). Dye-sensitized nanoparticles for heterogeneous photocatalysis: Cases studies with TiO2, ZnO, fullerene and graphene for water purification. Dyes Pigments.

[70] Martynov A.G., Safonova E.A., Tsivadze A.Y., Gorbunova Y.G. (2019). Functional molecular switches involving tetrapyrrolic macrocycles. Coord. Chem. Rev..

[71] Bettini S., Valli L., Giancane G. (2020). Applications of Photoinduced Phenomena in Supramolecularly Arranged Phthalocyanine Derivatives: A Perspective. Molecules.

[72] Calmeiro J.M.D., Tome J.P.C., Lourenco L.M.O. (2020). Supramolecular graphene-phthalocyanine assemblies for technological breakthroughs. J. Mater. Chem. C.

[73] Mongeon P., Paul-Hus A. (2016). The journal coverage of Web of Science and Scopus: A comparative analysis. Scientometrics.

[74] Zhu J.W., Liu W.S. (2020). A tale of two databases: The use of Web of Science and Scopus in academic papers. Scientometrics.

[75] Imahori H., Umeyama T., Kurotobi K., Takano Y. (2012). Self-assembling porphyrins and phthalocyanines for photoinduced charge separation and charge transport. Chem. Commun..

[76] Kerp H.R., Donker H., Koehorst R.B.M., Schaafsma T.J., van Faassen E.E. (1998). Exciton transport in organic dye layers for photovoltaic applications. Chem. Phys. Lett..

[77] Schwarze M., Tress W., Beyer B., Gao F., Scholz R., Poelking C., Ortstein K., Gunther A.A., Kasemann D., Andrienko D. (2016). Band structure engineering in organic semiconductors. Science.

[78] Capasso F. (1987). Band-Gap Engineering—From Physics and Materials to New Semiconductor-Devices. Science.

[79] Poelking C., Andrienko D. (2015). Design Rules for Organic Donor-Acceptor Heterojunctions: Pathway for Charge Splitting and Detrapping. J. Am. Chem. Soc..

[80] Ke L.L., Min J., Adam M., Gasparini N., Hou Y., Perea J.D., Chen W., Zhang H., Fladischer S., Sale A.C. (2016). A Series of Pyrene-Substituted Silicon Phthalocyanines as Near-IR Sensitizers in Organic Ternary Solar Cells. Adv. Energy Mater..

[81] Hu M., Bi C., Yuan Y.B., Bai Y., Huang J.S. (2016). Stabilized Wide Bandgap MAPbBr(x)I(3-x) Perovskite by Enhanced Grain Size and Improved Crystallinity. Adv. Sci..

[82] Aharon S., El Cohen B., Etgar L. (2014). Hybrid Lead Halide Iodide and Lead Halide Bromide in Efficient Hole Conductor Free Perovskite Solar Cell. J. Phys. Chem. C.

[83] Stranks S.D., Eperon G.E., Grancini G., Menelaou C., Alcocer M.J.P., Leijtens T., Herz L.M., Petrozza A., Snaith H.J. (2013). Electron-Hole Diffusion Lengths Exceeding 1 Micrometer in an Organometal Trihalide Perovskite Absorber. Science.

[84] Saliba M. (2018). Perovskite solar cells must come of age. Science.

[85] Berhe T.A., Su W.N., Chen C.H., Pan C.J., Cheng J.H., Chen H.M., Tsai M.C., Chen L.Y., Dubale A.A., Hwang B.J. (2016). Organometal halide perovskite solar cells: Degradation and stability. Energy Environ. Sci..

[86] Divitini G., Cacovich S., Matteocci F., Cina L., Di Carlo A., Ducati C. (2016). In situ observation of heat-induced degradation of perovskite solar cells. Nat. Energy.

[87] Suzuki A., Kida T., Takagi T., Oku T. (2016). Effects of hole-transporting layers of perovskite-based solar cells. Jpn. J. Appl. Phys..

[88] Swetha T., Singh S.P. (2015). Perovskite solar cells based on small molecule hole transporting materials. J. Mater. Chem. A.

[89] Guo J.J., Bai Z.C., Meng X.F., Sun M.M., Song J.H., Shen Z.S., Ma N., Chen Z.L., Zhang F. (2017). Novel dopant-free metallophthalocyanines based hole transporting materials for perovskite solar cells: The effect of core metal on photovoltaic performance. Sol. Energy.

[90] Guo J.J., Meng X.F., Niu J., Yin Y., Han M.M., Ma X.H., Song G.S., Zhang F. (2016). A novel asymmetric phthalocyanine-based hole transporting material for perovskite solar cells with an open-circuit voltage above 1.0 V. Synth. Met..

[91] Guo J.J., Meng X.F., Zhu H.W., Sun M.M., Wang Y.B., Wang W.N., Xing M.Y., Zhang F. (2019). Boosting the performance and stability of perovskite solar cells with phthalocyanine-based dopant-free hole transporting materials through core metal and peripheral groups engineering. Org. Electron..

[92] Kim Y.C., Yang T.Y., Jeon N.J., Im J., Jang S., Shin T.J., Shin H.W., Kim S., Lee E., Kim S. (2017). Engineering interface structures between lead halide perovskite and copper phthalocyanine for efficient and stable perovskite solar cells. Energy Environ. Sci..

[93] Cho K.T., Trukhina O., Roldan-Carmona C., Ince M., Gratia P., Grancini G., Gao P., Marszalek T., Pisula W., Reddy P.Y. (2017). Molecularly Engineered Phthalocyanines as Hole-Transporting Materials in Perovskite Solar Cells Reaching Power Conversion Efficiency of 17.5%. Adv. Energy Mater..

[94] Jiang X.Q., Yu Z., Lai J.B., Zhang Y.C., Hu M.W., Lei N., Wang D.P., Yang X.C., Sun L.C. (2017). Interfacial Engineering of Perovskite Solar Cells by Employing a Hydrophobic Copper Phthalocyanine Derivative as Hole-Transporting Material with Improved Performance and Stability. ChemSusChem.

[95] Zhang F.G., Yang X.C., Cheng M., Wang W.H., Sun L.C. (2016). Boosting the efficiency and the stability of low cost perovskite solar cells by using CuPc nanorods as hole transport material and carbon as counter electrode. Nano Energy.

[96] Liu Z.Y., Sun B., Liu X.Y., Han J.H., Ye H.B., Tu Y.X., Chen C., Shi T.L., Tang Z.R., Liao G.L. (2018). 15% efficient carbon based planar-heterojunction perovskite solar cells using a TiO2/SnO2 bilayer as the electron transport layer. J. Mater. Chem. A.

[97] Liu X.Y., Liu Z.Y., Sun B., Tan X.H., Ye H.B., Tu Y.X., Shi T.L., Tang Z.R., Liao G.L. (2018). 17.46% efficient and highly stable carbon-based planar perovskite solar cells employing Ni-doped rutile TiO2 as electron transport layer. Nano Energy.

[98] Ye H.B., Liu Z.Y., Liu X.Y., Sun B., Tan X.H., Tu Y.X., Shi T.L., Tang Z.R., Liao G.L. (2019). 17.78% efficient low-temperature carbon-based planar perovskite solar cells using Zn-doped SnO2 electron transport layer. Appl. Surf. Sci..

[99] Smecca E., Numata Y., Deretzis I., Pellegrino G., Boninelli S., Miyasaka T., La Magna A., Alberti A. (2016). Stability of solution-processed MAPbI(3) and FAPbI(3) layers. Phys. Chem. Chem. Phys..

[100] Saliba M., Matsui T., Domanski K., Seo J.Y., Ummadisingu A., Zakeeruddin S.M., Correa-Baena J.P., Tress W.R., Abate A., Hagfeldt A. (2016). Incorporation of rubidium cations into perovskite solar cells improves photovoltaic performance. Science.

[101] Zhao X., Kim H.S., Seo J.Y., Park N.G. (2017). Effect of Selective Contacts on the Thermal Stability of Perovskite Solar Cells. ACS Appl. Mater. Interfaces.

[102] Malinauskas T., Tomkute-Luksiene D., Sens R., Daskeviciene M., Send R., Wonneberger H., Jankauskas V., Bruder I., Getautis V. (2015). Enhancing Thermal Stability and Lifetime of Solid-State Dye-Sensitized Solar Cells via Molecular Engineering of the Hole-Transporting Material Spiro-OMeTAD. ACS Appl. Mater. Interfaces.

[103] Molina-Ontoria A., Zimmermann I., Garcia-Benito I., Gratia P., Roldan-Carmona C., Aghazada S., Graetzel M., Nazeeruddin M.K., Martin N. (2016). Benzotrithiophene-Based Hole-Transporting Materials for 18.2% Perovskite Solar Cells. Angew. Chem. Int. Ed..

[104] Huang C.Y., Fu W.F., Li C.Z., Zhang Z.Q., Qiu W.M., Shi M.M., Heremans P., Jen A.K.Y., Chen H.Z. (2016). Dopant-Free Hole-Transporting Material with a C-3h Symmetrical Truxene Core for Highly Efficient Perovskite Solar Cells. J. Am. Chem. Soc..

[105] Zhang J.B., Hua Y., Xu B., Yang L., Liu P., Johansson M.B., Vlachopoulos N., Kloo L., Boschloo G., Johansson E.M.J. (2016). The Role of 3D Molecular Structural Control in New Hole Transport Materials Outperforming Spiro-OMeTAD in Perovskite Solar Cells. Adv. Energy Mater..

[106] Zhou H.P., Chen Q., Li G., Luo S., Song T.B., Duan H.S., Hong Z.R., You J.B., Liu Y.S., Yang Y. (2014). Interface engineering of highly efficient perovskite solar cells. Science.

[107] Gao X.F., Li J.Y., Baker J., Hou Y., Guan D.S., Chen J.H., Yuan C. (2014). Enhanced photovoltaic performance of perovskite CH3NH3PbI3 solar cells with freestanding TiO2 nanotube array films. Chem. Commun..

[108] Novoselov K.S., Mishchenko A., Carvalho A., Neto A.H.C. (2016). 2D materials and van der Waals heterostructures. Science.

[109] Huang Y.L., Zheng Y.J., Song Z.B., Chi D.Z., Wee A.T.S., Quek S.Y. (2018). The organic-2D transition metal dichalcogenide heterointerface. Chem. Soc. Rev..

[110] Wang H.M., Li C.H., Fang P.F., Zhang Z.L., Zhang J.Z. (2018). Synthesis, properties, and optoelectronic applications of two-dimensional MoS_2_ and MoS_2_-based heterostructures. Chem. Soc. Rev..

[111] Choi J., Zhang H.Y., Choi J.H. (2016). Modulating Optoelectronic Properties of Two Dimensional Transition Metal Dichalcogenide Semiconductors by Photoinduced Charge Transfer. ACS Nano.

[112] Kafle T.R., Kattel B., Lane S.D., Wang T., Zhao H., Chan W.L. (2017). Charge Transfer Exciton and Spin Flipping at Organic Transition-Metal Dichalcogenide Interfaces. ACS Nano.

[113] Kafle T.R., Kattel B., Yao P., Zereshki P., Zhao H., Chan W.L. (2019). Effect of the Interfacial Energy Landscape on Photoinduced Charge Generation at the ZnPc/MoS_2_ Interface. J. Am. Chem. Soc..

[114] Huang Y., Zhuge F.W., Hou J.X., Lv L., Luo P., Zhou N., Gan L., Zhai T.Y. (2018). Van der Waals Coupled Organic Molecules with Monolayer MoS_2_ for Fast Response Photodetectors with Gate-Tunable Responsivity. ACS Nano.

[115] Lopez-Sanchez O., Lembke D., Kayci M., Radenovic A., Kis A. (2013). Ultrasensitive photodetectors based on monolayer MoS_2_. Nat. Nanotechnol..

[116] Nguyen E.P., Carey B.J., Harrison C.J., Atkin P., Berean K.J., Della Gaspera E., Ou J.Z., Kaner R.B., Kalantar-Zadeh K., Daeneke T. (2016). Excitation dependent bidirectional electron transfer in phthalocyanine-functionalised MoS_2_ nanosheets. Nanoscale.

[117] Park J.H., Sanne A., Guo Y.Z., Amani M., Zhang K.H., Movva H.C.P., Robinson J.A., Javey A., Robertson J., Banerjee S.K. (2017). Defect passivation of transition metal dichalcogenides via a charge transfer van der Waals interface. Sci. Adv..

[118] Canton-Vitoria R., Gobeze H.B., Blas-Ferrando V.M., Ortiz J., Jang Y., Fernandez-Lazaro F., Sastre-Santos A., Nakanishi Y., Shinohara H., D’Souza F. (2019). Excited-State Charge Transfer in Covalently Functionalized MoS_2_ with a Zinc Phthalocyanine Donor-Acceptor Hybrid. Angew. Chem. Int. Ed..

[119] Baeg K.J., Binda M., Natali D., Caironi M., Noh Y.Y. (2013). Organic Light Detectors: Photodiodes and Phototransistors. Adv. Mater..

[120] Qian C., Sun J., Kong L.A., Fu Y., Chen Y., Wang J.X., Wang S.T., Xie H.P., Huang H., Yang J.L. (2017). Multilevel Nonvolatile Organic Photomemory Based on Vanadyl-Phthalocyanine/para-Sexiphenyl Heterojunctions. ACS Photonics.

[121] Qian C., Sun J., Kong L.A., Gou G.Y., Zhu M.L., Yuan Y.B., Huang H., Gao Y.L., Yang J.L. (2017). High-Performance Organic Heterojunction Phototransistors Based on Highly Ordered Copper Phthalocyanine/para-Sexiphenyl Thin Films. Adv. Funct. Mater..

[122] Bian J., Feng J.N., Zhang Z.Q., Li Z.J., Zhang Y.H., Liu Y.D., Ali S., Qu Y., Bai L.L., Xie J.J. (2019). Dimension-Matched Zinc Phthalocyanine/BiVO4 Ultrathin Nanocomposites for CO2 Reduction as Efficient Wide-Visible-Light-Driven Photocatalysts via a Cascade Charge Transfer. Angew. Chem. Int. Ed..

[123] Prajapati P.K., Singh H., Yadav R., Sinha A.K., Szunerits S., Boukherroub R., Jain S.L. (2019). Core-shell Ni/NiO grafted cobalt (II) complex: An efficient inorganic nanocomposite for photocatalytic reduction of CO2 under visible light irradiation. Appl. Surf. Sci..

[124] Kumar A., Prajapati P.K., Aathira M.S., Bansiwal A., Boukherroub R., Jain S.L. (2019). Highly improved photoreduction of carbon dioxide to methanol using cobalt phthalocyanine grafted to graphitic carbon nitride as photocatalyst under visible light irradiation. J. Colloid Interface Sci..

[125] Roy S., Reisner E. (2019). Visible-Light-Driven CO2 Reduction by Mesoporous Carbon Nitride Modified with Polymeric Cobalt Phthalocyanine. Angew. Chem. Int. Ed..

[126] Corrigan N., Xu J.T., Boyer C. (2016). A Photoinitiation System for Conventional and Controlled Radical Polymerization at Visible and NIR Wavelengths. Macromolecules.

[127] Breloy L., Brezova V., Blacha-Grzechnik A., Presset M., Yildirim M.S., Yilmaz I., Yagci Y., Versace D.L. (2020). Visible Light Anthraquinone Functional Phthalocyanine Photoinitiator for Free-Radical and Cationic Polymerizations. Macromolecules.

[128] Xiao P., Dumur F., Graff B., Fouassier J.P., Gigmes D., Lalevee J. (2013). Cationic and Thiol-Ene Photopolymerization upon Red Lights Using Anthraquinone Derivatives as Photoinitiators. Macromolecules.

[129] Zhang J., Hill N., Lalevee J., Fouassier J.P., Zhao J.C., Graff B., Schmidt T.W., Kable S.H., Stenzel M.H., Coote M.L. (2018). Multihydroxy-Anthraquinone Derivatives as Free Radical and Cationic Photoinitiators of Various Photopolymerizations under Green LED. Macromol. Rapid Commun..

[130] Wang Z.H., Roffey A., Losantos R., Lennartson A., Jevric M., Petersen A.U., Quant M., Dreos A., Wen X., Sampedro D. (2019). Macroscopic heat release in a molecular solar thermal energy storage system. Energy Environ. Sci..

[131] Calvete M.J.F., Piccirillo G., Vinagreiro C.S., Pereira M.M. (2019). Hybrid materials for heterogeneous photocatalytic degradation of antibiotics. Coord. Chem. Rev..

[132] Lu W.Y., Xu T.F., Wang Y., Hu H.G., Li N., Jiang X.M., Chen W.X. (2016). Synergistic photocatalytic properties and mechanism of g-C3N4 coupled with zinc phthalocyanine catalyst under visible light irradiation. Appl. Catal. B Environ..

[133] Xu T.F., Ni D.J., Chen X., Wu F., Ge P.F., Lu W.Y., Hu H.G., Zhu Z.X., Chen W.X. (2016). Self-floating graphitic carbon nitride/zinc phthalocyanine nanofibers for photocatalytic degradation of contaminants. J. Hazard. Mater..

[134] Xu T.F., Wang D.N., Dong L.L., Shen H.B., Lu W.Y., Chen W.X. (2019). Graphitic carbon nitride co-modified by zinc phthalocyanine and graphene quantum dots for the efficient photocatalytic degradation of refractory contaminants. Appl. Catal. B Environ..

[135] Dong L.L., Xu T.F., Chen W.X., Lu W.Y. (2019). Synergistic multiple active species for the photocatalytic degradation of contaminants by imidazole-modified g-C3N4 coordination with iron phthalocyanine in the presence of peroxymonosulfate. Chem. Eng. J..

[136] Liang Q., Zhang M., Zhang Z.H., Liu C.H., Xu S., Li Z.Y. (2017). Zinc phthalocyanine coupled with UIO-66 (NH2) via a facile condensation process for enhanced visible-light-driven photocatalysis. J. Alloys Compd..

[137] Liu C., Li Y.H., Duan Q. (2020). Preparation of magnetic and thermal dual-responsive zinc-tetracarboxyl-phthalocyanine-g-Fe3O4@SiO2@TiO2-g-poly(N-isopropyl acrylamide) core-shell green photocatalyst. Appl. Surf. Sci..

[138] Tong J.T., Wang D.L., Wang D.C., Xu F., Duan R.M., Zhang D.F., Fan J., Dong B. (2020). Visible-Light-Driven Water-Fueled Ecofriendly Micromotors Based on Iron Phthalocyanine for Highly Efficient Organic Pollutant Degradation. Langmuir.

[139] Dini D., Calvete M.J.F., Hanack M., Chen W.Z., Ji W. (2006). Synthesis of axially substituted gallium, indium and thallium phthalocyanines with nonlinear optical properties. Arkivoc.

[140] Carvalho E.F.A., Calvete M.J.F., Cavaleiro J.A.S., Dini D., Meneghetti M., Tome A.C. (2010). Synthesis and high ranked NLT properties of new sulfonamide-substituted indium phthalocyanines. Inorg. Chim. Acta.

[141] Chen Y.X., Cao W., Wang C.M., Qi D.D., Wang K., Jiang J.Z. (2016). Four Dibutylamino Substituents Are Better Than Eight in Modulating the Electronic Structure and Third-Order Nonlinear-Optical Properties of Phthalocyanines. Inorg. Chem..

[142] Farinha A.S.F., Calvete M.J.F., Paz F.A.A., Tome A.C., Cavaleiro J.A.S., Sessler J.L., Tome J.P.C. (2014). Octatosylaminophthalocyanine: A reusable chromogenic anion chemosensor. Sens. Actuators B Chem..

[143] Carvalho E.F.A., Calvete M.J.F., Tome A.C., Cavaleiro J.A.S. (2009). Synthesis of sulfonamide-substituted phthalocyanines. Tetrahedron Lett..

[144] Bhattacharya S., Biswas C., Raavi S.S.K., Krishna J.V.S., Krishna N.V., Giribabu L., Soma V.R. (2019). Synthesis, Optical, Electrochemical, DFT Studies, NLO Properties, and Ultrafast Excited State Dynamics of Carbazole-Induced Phthalocyanine Derivatives. J. Phys. Chem. C.

[145] Zhao P., Wang Z.H., Chen J.S., Zhou Y., Zhang F.S. (2017). Nonlinear optical and optical limiting properties of polymeric carboxyl phthalocyanine coordinated with rare earth atom. Opt. Mater..

[146] Oluwole D.O., Yagodin A.V., Mkhize N.C., Sekhosana K.E., Martynov A.G., Gorbunova Y.G., Tsivadze A.Y., Nyokong T. (2017). First Example of Nonlinear Optical Materials Based on Nanoconjugates of Sandwich Phthalocyanines with Quantum Dots. Chem. Eur. J..

[147] Nwaji N., Jones B., Mack J., Oluwole D.O., Nyokong T. (2017). Nonlinear optical dynamics of benzothiazole derivatized phthalocyanines in solution, thin films and when conjugated to nanoparticles. J. Photochem. Photobiol. A.

[148] Nwaji N., Oluwole D.O., Mack J., Louzada M., Khene S., Britton J., Nyokong T. (2017). Improved nonlinear optical behaviour of ball type indium(III) phthalocyanine linked to glutathione capped nanoparticles. Dyes Pigments.

