# Supplementary material for: Phthalocyanines: An Old Dog Can Still Have New (Photo)Tricks!
Source: Molecules. 2021 May 10;26(9):2823. doi: 10.3390/molecules26092823 (PMC8126243; doi:10.3390/molecules26092823)
Supplement: Supplementary file 1 [file molecules-26-02823-s001.zip › molecules-1172215-supplementary.pdf]

Supplementary Material

Phthalocyanines: An Old Dog Can Still Have New (Photo)Tricks!

Andrea M. Schmidt [1] and Mário J. F. Calvete [2,*]

[1] LifeEstetika, Laser Solutions, Universitätstadt Tübingen, Maria-von-Linden Strasse, 72076 Tübingen, Germany; andrea.m.schmidt.dls@gmail.com
[2] University of Coimbra, CQC, Department of Chemistry, Rua Larga, 3004-535 Coimbra, Portugal
* Correspondence: mcalvete@qui.uc.pt

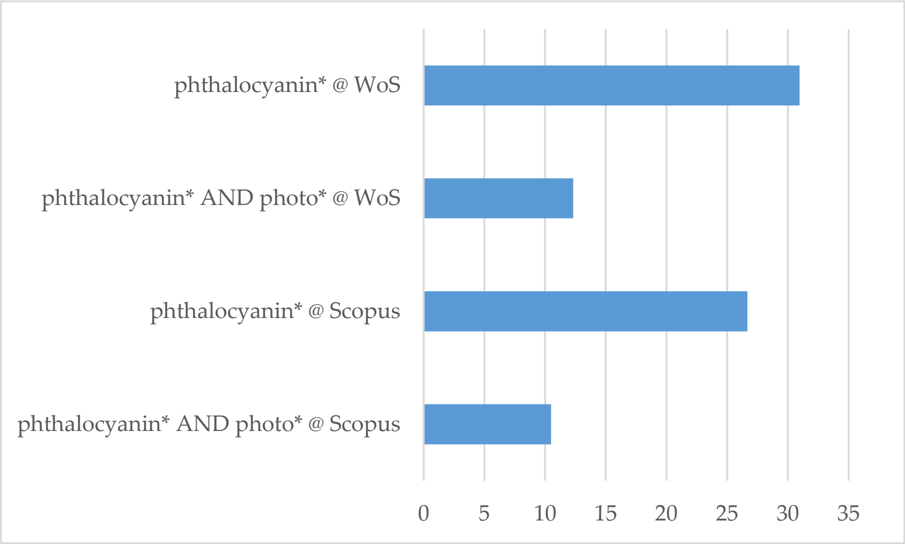

Figure S1. Results obtained using search strings (phthalocyanin*) and (phthalocyanin* AND photo*) present in the title and/or abstract and/or keywords in search engines Scopus® and Web of Science® (WoS). Results are in thousands.

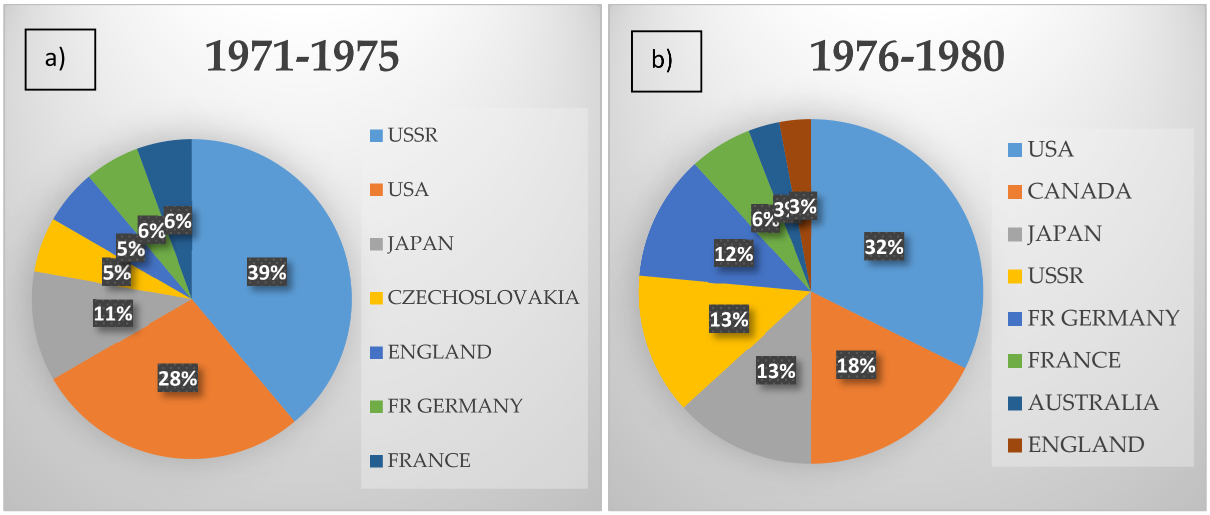

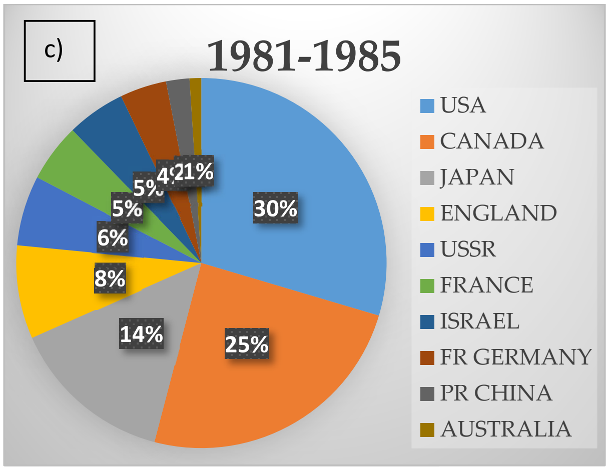
c)
1981-1985
USA
CANADA
JAPAN
ENGLAND
USSR
FRANCE
ISRAEL
FR GERMANY
PR CHINA
AUSTRALIA
30%
25%
14%
8%
6%
5%

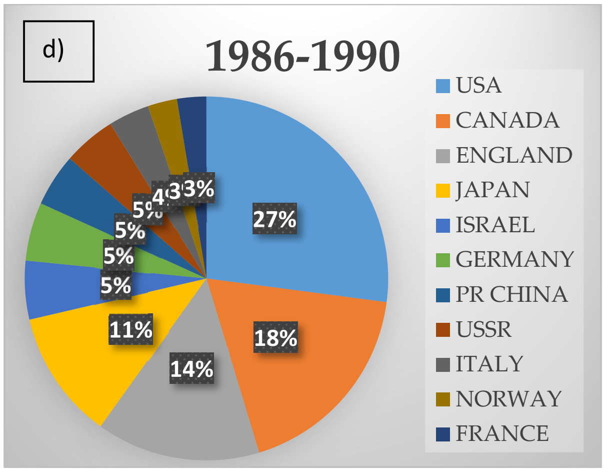
d)
1986-1990
USA
CANADA
ENGLAND
JAPAN
ISRAEL
GERMANY
PR CHINA
USSR
ITALY
NORWAY
FRANCE
27%
18%
14%
11%
5%
5%
5%

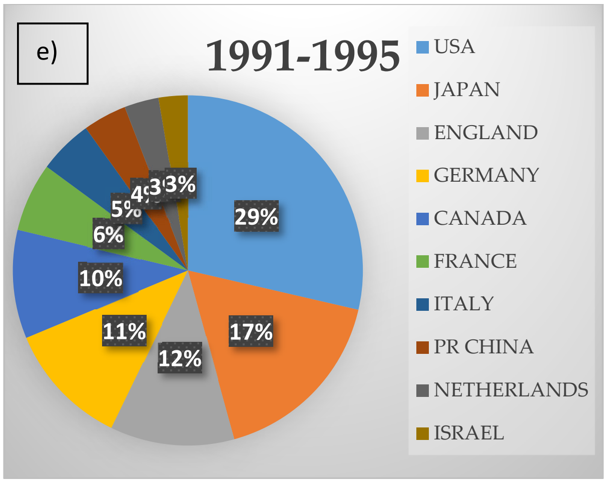
e)
1991-1995
USA
JAPAN
ENGLAND
GERMANY
CANADA
FRANCE
ITALY
PR CHINA
NETHERLANDS
ISRAEL
29%
17%
12%
11%
10%
6%

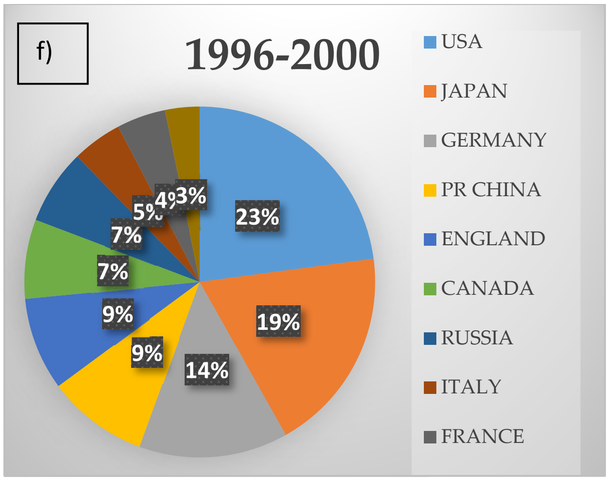
f)
1996-2000
USA
JAPAN
GERMANY
PR CHINA
ENGLAND
CANADA
RUSSIA
ITALY
FRANCE
23%
19%
14%
9%
9%
7%
7%

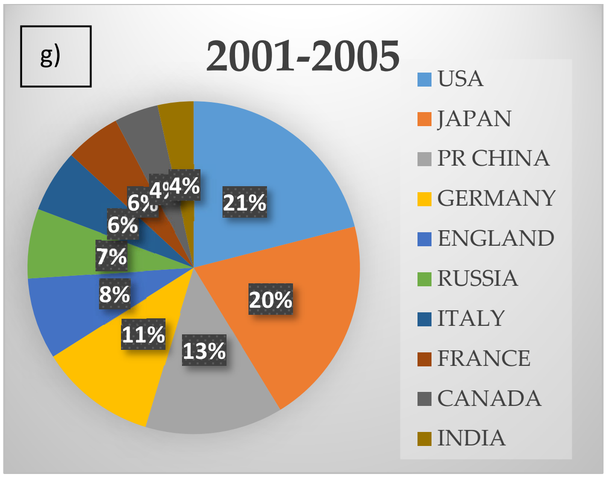
g)
2001-2005
USA
JAPAN
PR CHINA
GERMANY
ENGLAND
RUSSIA
ITALY
FRANCE
CANADA
INDIA
21%
20%
13%
11%
8%
7%
6%

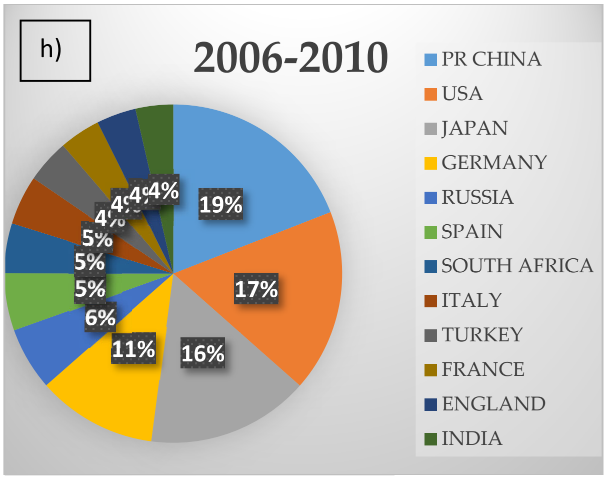
h)
2006-2010
PR CHINA
USA
JAPAN
GERMANY
RUSSIA
SPAIN
SOUTH AFRICA
ITALY
TURKEY
FRANCE
ENGLAND
INDIA
19%
17%
16%
11%
6%
5%
5%

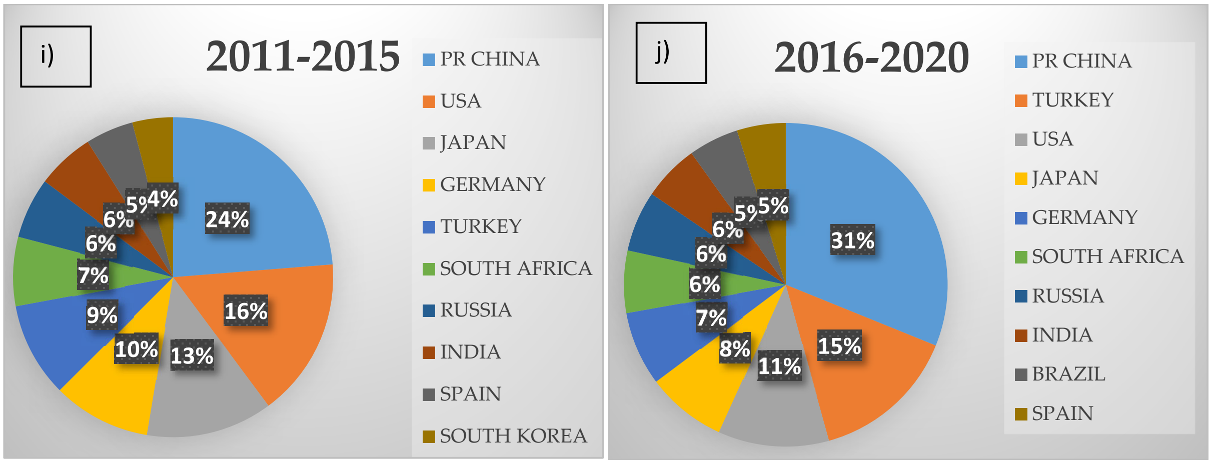

Figure S2. Country weight in publication of articles by 5-year span, from 1971 to 2020. Articles were found by using search string "phthalocyanin*" followed by refining with term "photo*". **a**) 1971-1975; **b**) 1976-1980; **c**) 1981-1985; **d**) 1986-1990; **e**) 1991-1995; **f**) 1996-2000; **g**) 2001-2005; **h**) 2006-2010; **i**) 2011-2015; **j**) 2016-2020.
